# Expectation and attention increase the integration of top-down and bottom-up signals in perception through different pathways

**DOI:** 10.1371/journal.pbio.3000233

**Published:** 2019-04-30

**Authors:** Noam Gordon, Naotsugu Tsuchiya, Roger Koenig-Robert, Jakob Hohwy

**Affiliations:** 1 Cognition and Philosophy Lab, Philosophy Department, Monash University, Clayton, Victoria, Australia; 2 Monash Institute of Cognitive and Clinical Neurosciences, Monash University, Clayton, Victoria, Australia; 3 School of Psychological Sciences, Monash University, Clayton, Victoria, Australia; 4 Center for Information and Neural Networks, National Institute of Information and Communications Technology, Suita, Osaka, Japan; 5 Advanced Telecommunications Research Computational Neuroscience Laboratories, Soraku-gun, Kyoto, Japan; 6 School of Psychology, The University of New South Wales, Sydney, New South Wales, Australia; University of Oregon, UNITED STATES

## Abstract

Perception likely results from the interplay between sensory information and top-down signals. In this electroencephalography (EEG) study, we utilised the hierarchical frequency tagging (HFT) method to examine how such integration is modulated by expectation and attention. Using intermodulation (IM) components as a measure of nonlinear signal integration, we show in three different experiments that both expectation and attention enhance integration between top-down and bottom-up signals. Based on a multispectral phase coherence (MSPC) measure, we present two direct physiological measures to demonstrate the distinct yet related mechanisms of expectation and attention, which would not have been possible using other amplitude-based measures. Our results link expectation to the modulation of descending signals and to the integration of top-down and bottom-up information at lower levels of the visual hierarchy. Meanwhile, the results link attention to the modulation of ascending signals and to the integration of information at higher levels of the visual hierarchy. These results are consistent with the predictive coding account of perception.

## Abstract 2

Perception is believed to result from the integration of sensory information with pre-existing information available in the brain. This study provides direct neurophysiological evidence showing how expectation and attention increase such integration of top-down and bottom-up neural signals in perception.

## Introduction

Perception is not a simple ‘bottom-up’ mechanism of progressive processing of the sensory input. Instead, perception is made possible by processing sensory information in the context of information spanning multiple cortical levels. For example, one way to understand the visual system’s ability to reach unambiguous representations from highly complex, variable, and inherently ambiguous sensory inputs is in terms of Bayesian inference and probabilistic integration of prior knowledge (top-down) with stimulus features (bottom-up). Crucially, within these kinds of frameworks, the integration of top-down and bottom-up signals is dynamically modulated by cognitive and potentially interacting factors such as expectation and attention [1–5].

While expectation and attention are much studied, attempts to dissociate the two and study their unique yet interrelated underlying mechanisms are relatively recent and far from complete [6–10]. Great caution in experimental design is required in order to obtain empirical data that permit a genuine distinction between the neural processes underlying expectation and attention. A fundamental challenge is to keep expectation and attention sufficiently separate; studies tend either to rely on explicit probability cues, which introduces task demands, or to vary stimulus properties across conditions, which can confound expectation and attention. The paradigms we introduce in this study were designed specifically to avoid such pitfalls.

The theoretical framework under which we consider the potentially distinct roles of expectation and attention in perception is that of hierarchical perceptual inference. Rather than focusing on simple feedforward architectures, this framework emphasises the role of recurrent networks in which signals propagate within and between hierarchical levels through bidirectional bottom-up (ascending) and top-down (descending) pathways. These ideas have been developed and formalised in various influential predictive coding models [2,5,11–13]. Here, we specifically appeal to the Bayesian version of predictive coding, in which the brain’s ability to infer the causes of its sensations is attributed to its ability to embody the statistical structure in the environment within a generative model describing the hierarchical and dynamic statistics of the external world [2,11]. Such models of predictive coding view perception as a process of inferring the causes and states in the external world that cause the sensory input. The sensory input, or sensory evidence, is modulated as it ascends through the sensory hierarchy. Under the predictive coding framework we appeal to here, expectations allow predictive signals to descend from higher to lower levels in the cortical hierarchy, at which they are used to filter the sensory evidence such that what remains of it as it ascends through the sensory hierarchy is conceived as prediction error—the gap between the descending predictions and the ascending sensory signals (see also Discussion in which we discuss additional models of predictive coding that propose alternative mechanisms of hierarchical filtering). The prediction errors, in turn, allow for the higher-level expectations and subsequent predictions to be optimised in an iterative fashion. The outcome of this hierarchical prediction error minimisation (resolution) process is, according to the predictive coding account described above, perception.

**Fig 1:**
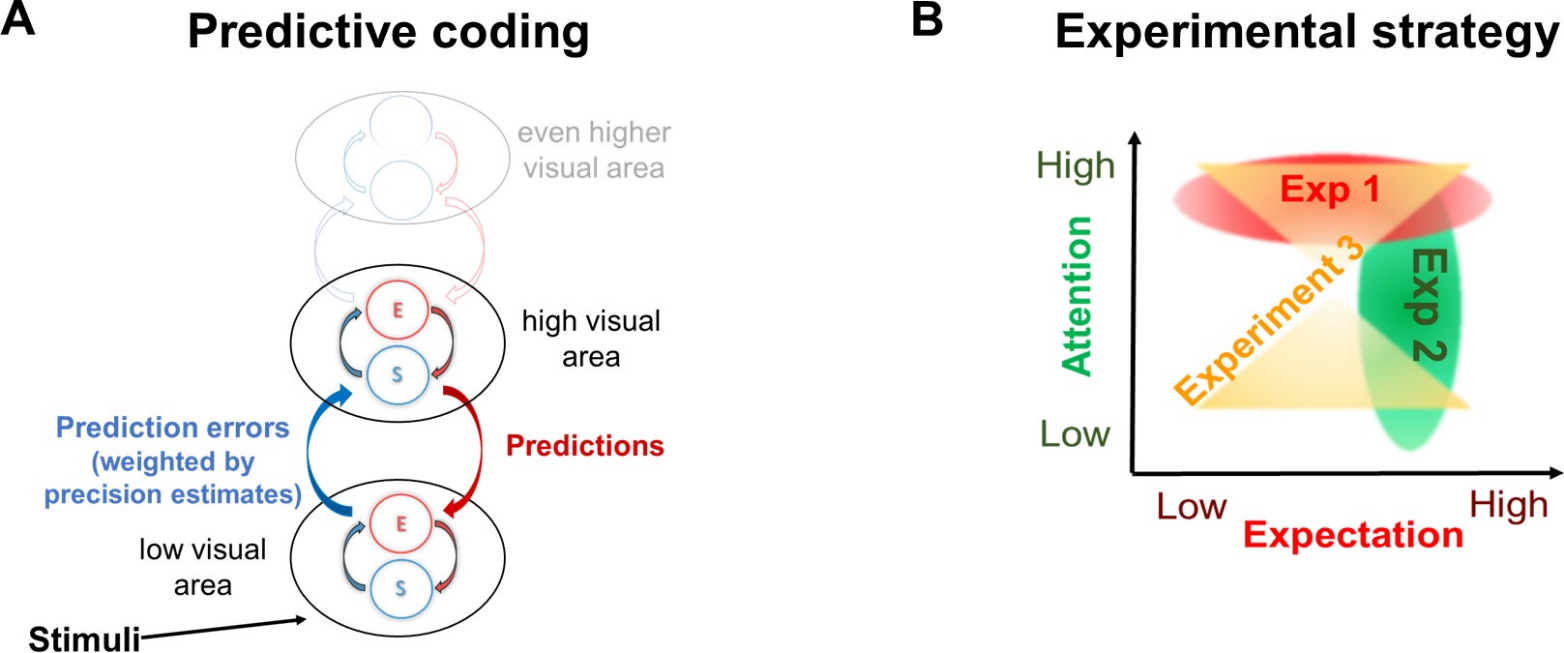
Theoretical background and experimental strategy. (A) The predictive coding theory of perception describes perception as the inference made about the state of the external world and the causes of the sensory input. In the predictive coding model we appeal to in this study, expectations allow predictive signals to descend from higher to lower levels in the cortical hierarchy, at which they are tested against sensory information. The discrepancy between the two—the prediction error—propagates up the hierarchy, allowing for the higher-level expectations and subsequent predictions to be optimised. Expectations and prediction errors are suggested to be coded within each hierarchical level by distinct neural populations referred to as S and E units, respectively [20,21]. Prediction errors are suggested to be weighted by their estimated (and expected) precision such that high precision estimates lead to enhancement of prediction error signals via synaptic gain mechanisms. (B) Conceptual figure of the three experiments used to comprehensively examine the role of expectation and attention in the integration of top-down and bottom-up signals. In Experiment 1 (red oval), expectation was manipulated while the visual stimuli were kept constant across conditions. In Experiment 2, attention was manipulated while expectation and the visual stimuli were held constant across conditions (green oval). In Experiment 3, we applied a novel analysis method to the data obtained in a previous study (22) in order to simultaneously examine main effects of expectation and attention as well as the interaction between the two (yellow hourglass shape). For consistency, all figures in Results use shades of red for expectation and shades of green for attention. E, error; S, state.

Prediction errors may result from two related sources: inaccurate top-down predictions (that do not match the actual state of the external environment) or imprecise or noisy bottom-up sensory information (such as vision in a foggy day or hearing through a brick wall). An efficient system should therefore incorporate an estimation of the precision (i.e., inverse of variance) of sensory signals. The more precise a prediction error is estimated to be, the more ‘reliable’ it is considered to be, leading to more revision of the generative model. While attention is a multifaceted mechanism related to perception and cognition and is yet to be fully understood, endogenous attention is broadly viewed as a top-down mechanism influencing the processing of bottom-up signals and perception [14]. This notion has been incorporated in various ways into different models of perception. For example, under the predictive coding model described above, some experimental evidence is consistent with the association of attention with the proposed mechanism for optimising precision estimates and weighting of prediction error (e.g., [15] in the visual domain and [16] in the auditory domain, though cf. [17,18]). In more mechanistic terms, attention may allow the ‘prioritisation’ of signals expected to be more precise by means of increasing the synaptic gain of neuronal units encoding precision estimates (Fig 1A) [3]. Expectation and attention therefore relate, under the predictive coding framework, to descending and ascending signals, respectively.

Several implications follow from this account of expectations, predictions, prediction error minimisation, and attention. On the one hand, highly predictable stimuli are expected to yield smaller prediction errors and thus attenuated prediction error–related neural activity. On the other hand, if attention estimates high precision of the signal (i.e., greater prediction error impact), then even expected stimuli will yield greater prediction error–related neural activity. Indeed, various studies suggest an interaction between attention and expectation and have demonstrated that when stimuli are unattended (e.g., they are task irrelevant), high levels of expectation can result in reduced sensory signals; however, when stimuli are attended (e.g., they are task relevant), expected stimuli can, in fact, result in greater neural activity ([19] but also see [17]).

The goal of the present study is to elucidate the mechanisms underlying attention and expectation and to better understand the relationship between these factors in perception. To do so, we performed three experiments and analyses aimed at comprehensively studying these factors while avoiding the potential pitfalls described above (Fig 1B).

**Fig 2:**
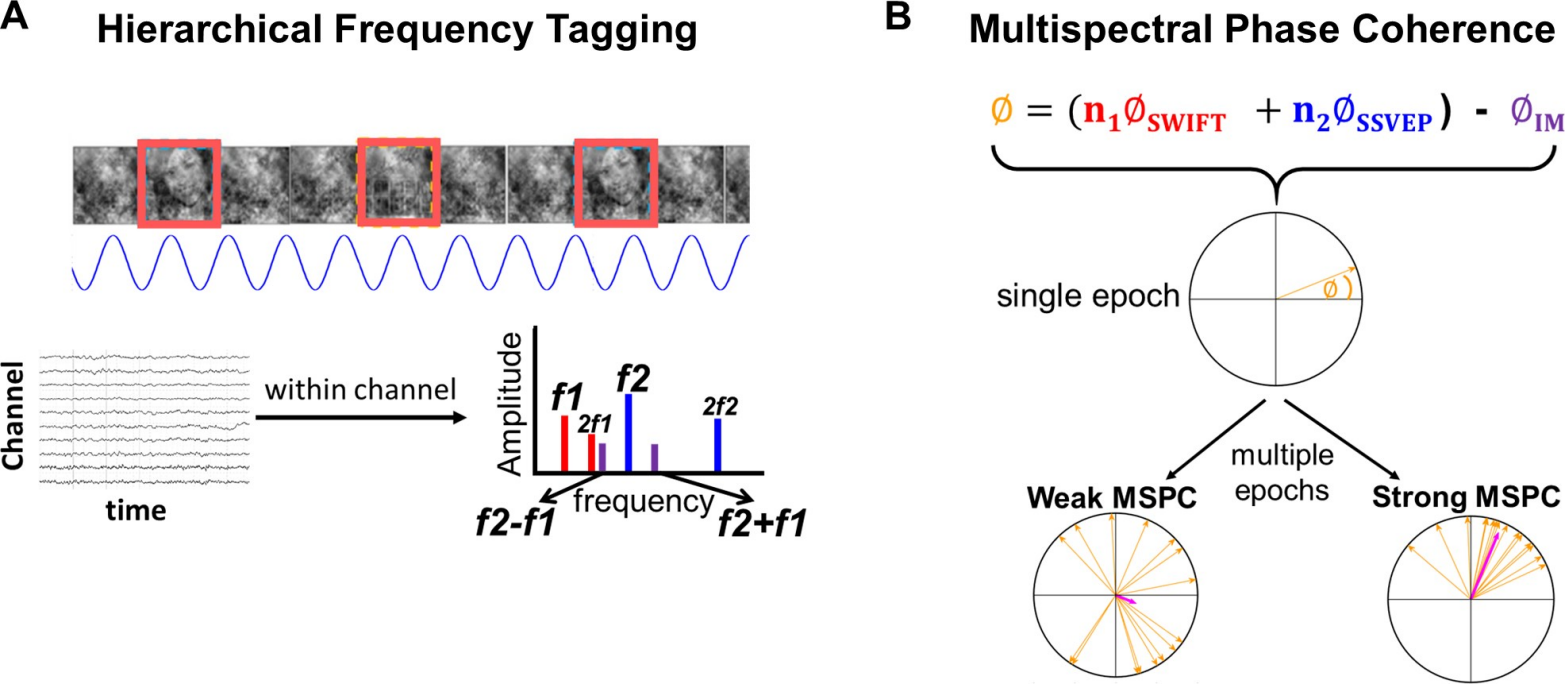
Stimulus construction and analysis methodologies. (A) All experiments implemented the HFT method using face and house images. SWIFT sequences are presented at given frequencies, allowing tagging of image-recognition activity (red rectangles). Contrast modulation is applied at a higher frequency, inducing SSVEP (blue sinusoid). When analysing the EEG data in the frequency domain (bottom graph with multiple peaks), peaks in the power spectrum can be seen at the fundamental frequencies and their harmonics (red bars for SWIFT f1 and blue bars for SSVEP f2). Additional peaks at IM components (e.g., purple bars for f2 + f1 and f2 − f1) are suggested to indicate integration of bottom-up SSVEP signals with top-down SWIFT signals. (B) The MSPC [36] quantifies the degree to which an IM frequency component is driven by the phases of the fundamental input frequencies. In other words, the degree to which the IM component reflects an interaction between those input frequencies. Within each epoch, we first calculate the difference between the sum of the (weighted) phases of the fundamental input frequencies and the phase of the IM component. Then, we compute the coherence of this value across multiple epochs applying the same method as in the well-known phase-locking value (see Methods for a detailed description.) Here, we introduced a novel distinction between two measures—MSPCstim and MSPCres—which differ in what we consider the ‘input’ signals to be. Specifically, the MSPCstim ties the IM phase to the phases of the stimulus itself (i.e., the images presented on the screen), while MSPCres ties it to the phases of the tagged neural responses (See Methods). We suggest that these measures distinguish between neural interactions occurring at lower and higher cortical levels, respectively. EEG, electroencephalography; HFT, hierarchical frequency tagging; IM, intermodulation; MSPCres, multispectral phase coherency (response); MSPCstim, MSPC (stimulus); SSVEP, steady-state visual evoked potentials; SWIFT, semantic wavelet-induced frequency-tagging.

All experiments utilised the hierarchical frequency–tagging (HFT) method in electroencephalography (EEG) ([22]; Fig 2A, S1 Video, and S2 Video). The HFT method was designed to investigate hierarchical visual processing. Its strength lies in its ability to distinguish between neural signals derived from different cortical levels while providing a measure for the integration of these signals. In brief, two frequency-tagging methods are combined: the steady-state visual evoked potentials (SSVEP, [23,24]) and the semantic wavelet-induced frequency-tagging (SWIFT, [25]). While SSVEP originates primarily in lower visual areas in the occipital cortex [26], SWIFT has been shown to selectively tag high-level object representation areas but not early visual areas in both EEG [25] and functional magnetic resonance imaging (fMRI) [27]. Given this evidence, we interpret the spectral power of the recorded signal at the tagged frequencies for the SSVEP and SWIFT as reflecting lower- and higher-level cortical activity, respectively.

Crucially, IM components, that is, linear combinations of the fundamental input frequencies, appear in the output of a system when the input frequencies interact or integrate nonlinearly within the system. As such, the IMs serve as a measure of nonlinear integration of input signals. While more theoretically committed notions of the term ‘integration’ can be found in the literature, we use it here to indicate that the output of the system is jointly determined by the different signals that feed into it. In the brain, nonlinear neural interactions enable rich, context-dependent information processing and play a key role in perception [28–30]. Indeed, several EEG studies have utilized IMs to reveal the mechanisms of visual object recognition ([31–33]; for a recent review of IMs in perception research, see [34]). Previously, the stimulus modulations used to elicit the SSVEP and the SWIFT responses were shown to tag activity at lower and at higher visual areas, respectively [27,35]. In the HFT method, we understand the integration of the signals tagged by the SSVEP and SWIFT frequencies, as manifested by the IMs, to reflect the integration of top-down SWIFT signals with bottom-up SSVEP signals. This interpretation was supported by our previous study [22] and further buttressed by the novel paradigms used here.

IMs can be quantified using amplitude-based measures, as long as the amplitudes of the fundamental frequencies do not confound the interpretation of the IMs (see [34] for details). Importantly, we introduce a novel distinction between two phase measures of the IM signal based on the multispectral phase coherence (MSPC, [36]). The first measure, MSPCstim, ties the IMs to the phases of the SWIFT and SSVEP stimulus modulation and the second one, MSPCres, to the tagged SWIFT and SSVEP neural response. We argue that these two phase-based temporal measures distinguish neural signal integration occurring at different hierarchical levels (Fig 2B and described in greater detail in Discussion and Methods). We hypothesised that if expectation and attention indeed relate to descending and ascending signals, respectively, their influence should manifest differently in these two measures.

Two new paradigms were designed to ensure two critical experimental aspects: that expectation and attention are manipulated individually with equivalent visual stimuli and that their modulatory effects are determined cognitively, minimising any difference in demand characteristics. In addition to these, new analyses performed on previously published data (Gordon and colleagues, 2017) allowed a direct examination not only of expectation and attention main effects but of the interaction between the two. Indeed, the two-phase measures we introduce here were modulated differently by expectation and attention, providing direct physiological evidence for their distinct hierarchical modulation of perceptual processing.

## Results

### Expectation: Experiment 1

#### Behavioral task

Experiment 1 manipulated expectation by using two tasks, specifically designed to avoid explicit indication of expectation levels in the instructions (thus minimising demand characteristics), while holding the visual stimuli equivalent across conditions. In each HFT trial, a series of consecutive SWIFT-scrambled sequences (of one house and one face image) were presented to participants (S3 Video) who were required to perform one of two tasks. In the image-repetition (IR) task, participants were requested to press the space bar when either image repeated itself either three or four times (as instructed before each trial). Stimuli in these trials were considered ‘unexpected’ as participants could not predict the upcoming image. In the pattern-violation (PV) tasks, participants were required to memorise a pattern of 5–6 images presented to them as text (‘Face, House …’ etc.) before the trial. When the trial began, the pattern repeated itself over and over, and participants were instructed to press the space bar when the pattern was violated. Thus, in PV trials, almost all upcoming images were highly predictable, so expectations were much more reliable than in the IR trials. Each series of face and house images (e.g., F, H, H, F, H, F, H, H, F, H…) appeared in one PV and in one IR trial (using different images), such that the series used for both tasks were, in fact, identical.

Participants were tested for four blocks, in the order of PV, IR, PV, and IR block. After completing all four blocks, participants were asked to compare the PV and the IR tasks and to report whether they noticed the underlying patterns in the IR tasks. Indeed, despite the PV blocks preceding their matching IR blocks (see Methods), only *N* = 3 out of 15 confirmed noticing an underlying pattern in a few IR trials. This validates our assumption that the PV and IR tasks manipulated the expectation for upcoming stimuli. Second, *N* = 10 out of 15 participants reported finding it more difficult to recognise the actual images of the IR compared to the PV trials. Several participants reported that the house and the face images tended to perceptually ‘blend’ more with each other in the IR trials. (Note that some elements of the images, such as edges with strong contrasts, may also remain visible to some degree in the ‘scrambled’ frames, accounting for why images could sometimes be perceived as blended; see S3 Video.). Given that the same method was used to construct all stimuli in the experiment, the reported differences in perception can be strictly attributed to the task instructions, highlighting the impact of one’s expectation on conscious perception.

### EEG analysis: Expectation modulates MSPCstim but not MSPCres

After applying the fast Fourier transform (FFT) on the EEG data of each trial, we verified that tagging was obtained for both the SWIFT and the SSVEP frequencies (SWIFT = f1 = 1.2 Hz and SSVEP = f2 = 15 Hz). Peak amplitude signal-to-noise ratios (SNRs) at both fundamental frequencies and their harmonics were evident in the FFT spectrum averaged across all electrodes, trials, and participants (S1 Fig, top row).

Critically, for the purpose of this paper, we examined the effect of expectation (predictability) on the IM signals. Based on the MSPC, we quantified the degree to which the IM phases were driven by the SWIFT and SSVEP phases. As detailed in Methods, we introduce a novel distinction between the MSPCstim measure in which the stimulus (image) SWIFT and SSVEP phases are considered as the inputs, and the MSPCres measure in which the EEG SWIFT and SSVEP neural response phases are considered as the inputs (both MSPCstim and MSPCres were computed individually within each electrode). As detailed in Discussion, we suggest these measures to indicate cortical signal integration occurring at different levels.

**Fig 3:**
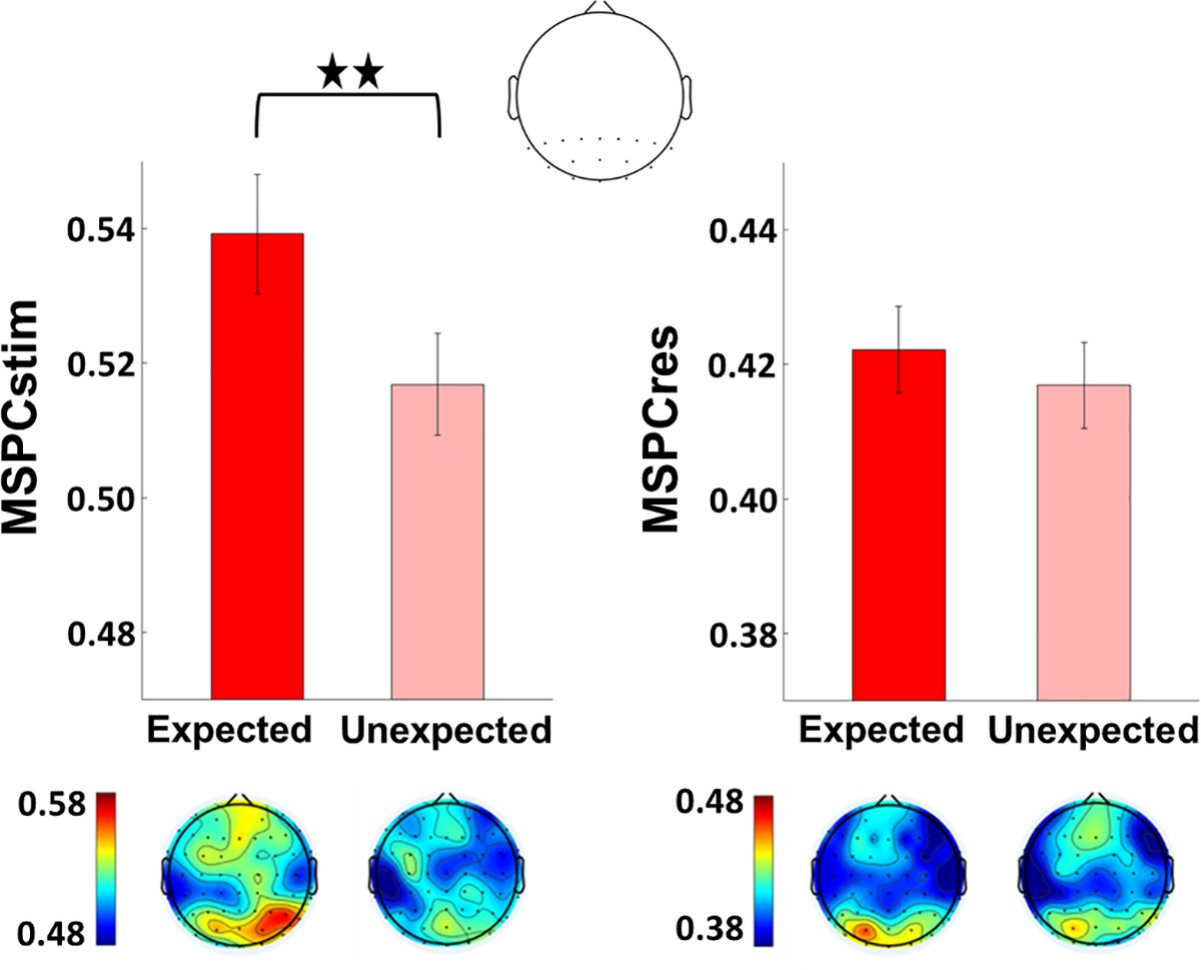
Expectation modulates MSPCstim but not MSPCres. MSPC averaged across the two second-order IM components (f1 + f2 and f1 − f2) in the expected and the unexpected conditions. Results are shown for a posterior ROI (17 electrodes, top) and the scalp topographies (bottom). Error bars represent standard error across subjects (*N* = 15). The MSPCstim measure (left) quantifies IM responses by examining the degree to which the IM phase is driven by the phases of SWIFT and SSVEP stimulus (image) modulation. Conversely, the MSPCres measure (right) examines the degree to which the IM phase is driven by the tagged SWIFT and SSVEP neural response phases. These measures are therefore suggested to indicate signal integration occurring at earlier and at later stages of cortical processing, respectively. MSPCstim (left) was higher for the PV (expected) trials compared to the IR (unexpected) trials (χ2 = 22.9, *P* < 0.001), indicating increased neural integration between the SWIFT and SSVEP signals when stimuli are expected. This effect was not evident for the MSPCres measure (χ2 = 1.36, *P* > 0.05). The data underlying this figure is available in FigShare at DOI: 10.26180/5b9abfe5687e3. IM, intermodulation; MSPCres, multispectral phase coherency (response); MSPCstim, MSPC (stimulus); SSVEP, steady-state visual evoked potentials; SWIFT, semantic wavelet-induced frequency-tagging.

MSPC values were calculated individually for each channel (with reference to its own SSVEP and SWIFT phases) within each trial (see Methods). Analyses were then performed on the average of both second-order IM components (f2 − f1 = 13.8 Hz and f2 + f1 = 16.2 Hz) in a posterior region-of-interest (ROI) (17 electrodes), including all occipital (Oz, O1, and O2), parieto–occipital (POz, PO3–PO4, and PO7–PO8), and parietal (Pz and P1–P8) electrodes. As shown in Fig 3, MSPCstim was higher for the PV (expected) trials compared to the IR (unexpected) trials (χ2 = 22.9, *P* < 0.001), indicating increased neural integration between the SWIFT and SSVEP signals when stimuli are expected. This effect was not evident for the MSPCres measure (χ2 = 1.36, *P* > 0.05). The significance of the MSPCstim versus MSPCres result will be discussed later when comparing the relationship between these measures across all experiments and analyses.

### Attention: Experiment 2

#### Behavioral task

In Experiment 2, HFT trials with house and face images were presented to participants with contrast modulation (SSVEP) at 12 Hz. Unlike Experiment 1, the two images used within each trial (one house and one face image) were presented using two different SWIFT frequencies (0.8 Hz and 1 Hz, counterbalanced across trials). Importantly, images were superimposed via alpha blending, enabling simultaneous tagging of both frequencies (one for each image type) within each trial. In each trial, participants were requested to count either the faces or the houses or to perform a demanding central-attention task (see further details in Methods). These behavioural tasks defined each image as either ‘attended’ or ‘unattended’ within each trial. Note that only 70%–85% of the SWIFT cycles contained the face or house images while the rest of the cycles contained their matching ‘noise’ sequences (See Methods). This ensured that the attentional task was sufficiently demanding (S4 Video).

### EEG analysis: Attention modulates MSPCres but not MSPCstim

First, we verified that we were able to obtain separate tagging for the relevant frequencies (two SWIFT: 0.8 Hz and 1 Hz and SSVEP: 12 Hz). Indeed, peak amplitude SNRs at all three fundamental frequencies were evident in the FFT spectrum averaged across all electrodes, trials, and participants (S1 Fig, bottom row).

**Fig 4:**
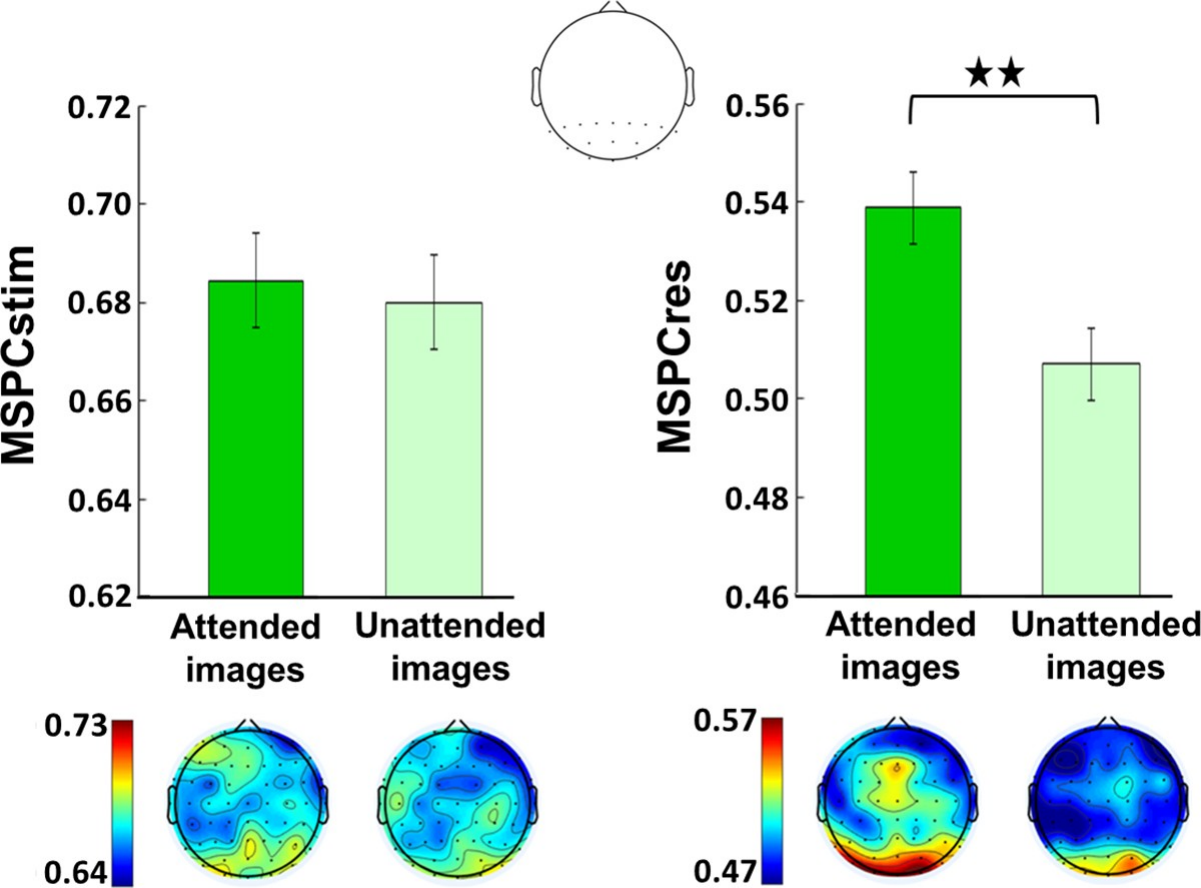
Attention modulates MSPCres but not MSPCstim. MSPC averaged across the two second-order IM components of the attended and the unattended images. (Note that within each trial, different SWIFT frequencies were used for the attended and the unattended images, each resulting in a different set of second-order IMs.) Results are shown for a posterior ROI (17 electrodes, top) and the scalp topographies (bottom). Error bars represent standard error across subjects (*N* = 11). The MSPCstim measure (left) quantifies IM responses by examining the degree to which the IM phase is driven by the phases of the SWIFT and SSVEP stimulus (image) modulations. Conversely, the MSPCres measure (right) examines the degree to which the IM phase is driven by the tagged SWIFT and SSVEP neural response phases. MSPCres (right) was higher for the attended compared to the unattended images (χ2 = 41.4, *P* < 0.001), indicating increased neural integration between the SWIFT and SSVEP signals when stimuli are attended. This effect was not evident for the MSPCstim measure (χ2 = 1.21, *P* > 0.05). The data underlying this figure is available in FigShare at DOI: 10.26180/5b9abfe5687e3. IM, intermodulation; MSPCres, multispectral phase coherency (response); MSPCstim, MSPC (stimulus); ROI, region of interest; SSVEP, steady-state visual evoked potentials; SWIFT, semantic wavelet-induced frequency-tagging.

As in Experiment 1, MSPC values were calculated individually for each channel within each trial (see Methods) and statistical analyses were performed on the average of both second-order IM components in a posterior ROI. (Note that in Experiment 2, there were two second-order IMs for the attended SWIFT stimuli and another two distinct second-order IMs for the unattended SWIFT stimuli. For example, if the attended stimuli appeared at 0.8 Hz and the unattended at 1 Hz, then the 12.8 Hz and 11.2 Hz IMs would relate to the attended stimuli while the 13 Hz and 11 Hz IMs would relate to the unattended stimuli.) Interestingly, as opposed to results from Experiment 1, the effect of attention was evident in the MSPCres measure but not MSPCstim (Fig 4). Specifically, MSPCSres was higher for the attended compared to the unattended images (χ2 = 41.4, *P* < 0.001). This effect was not evident for the MSPCstim measure (χ2 = 1.21, *P* > 0.05).

Further analysis of the data from Experiments 1 and 2 show comparable results for various higher-order IMs and is consistent with the suggestion that the attentional modulation manifests at higher cortical levels (S4 Text and S2 Fig). In brief, the effect of attention was most notable for the fourth order IMs (2f2 ± 2f1), and additional analysis suggests this effect to reflect interactions occurring at later rather than earlier processing stages.

### Interaction of attention and expectation: Reanalysis of previous study

After differentiating expectation and attention in Experiments 1 and 2 and establishing that both expectation and attention are associated with enhanced IMs, we returned to our previously published data [22] to examine the interaction between these factors and to evaluate the consistency of the results from that study with those of Experiment 1 and Experiment 2 here (S2 Text). In the previous study, house and face SWIFT cycles were presented in each trial in a pseudorandom order, and participants were asked to count either the houses or the faces. Certainty (expectation) levels were categorised based on the proportion of house and face images appearing in each trial. The behavioural task was introduced to ensure participants were engaged with the task, yet it also introduced a within-trial difference between the attended (counted) and unattended images, which was not analysed in that study. Here, we added an additional attention variable allowing examination of the interaction between expectation and attention (see Methods).

**Fig 5:**
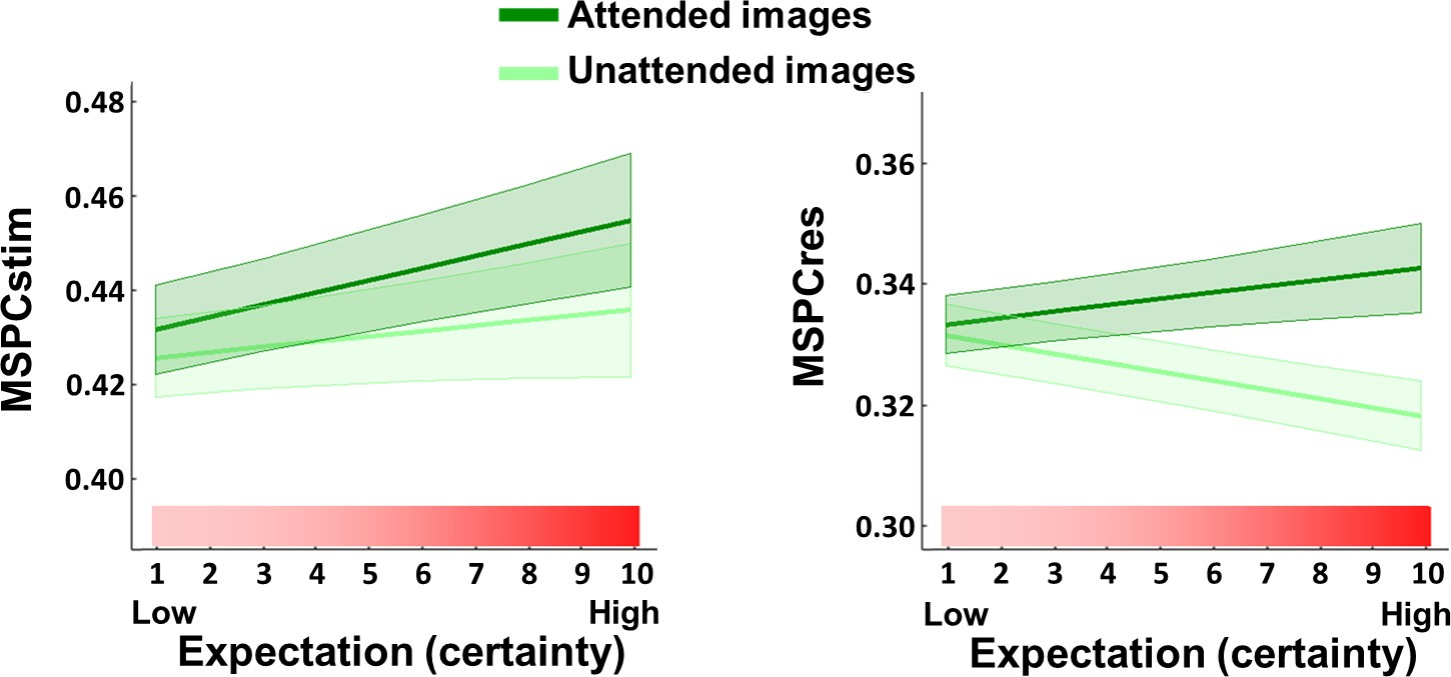
The expectation–attention interaction is significant for MSPCres but not MSPCstim. Predicted MSPCstim (left) and MSPCres (right) values obtained from a full Linear Mixed Effects interaction model, with their standard error indicated by the shaded area. The model included expectation, attention, and an expectation–attention interaction term as the fixed effects, while the random effects included a random intercept for frequency nested within channels nested within participants and random expectation and attention slopes for each participant. Consistent with the colours used in the previous figures, attended images are represented by the dark green lines and unattended images by the light green lines, while the pink–red gradient indicates increasing expectation. The significance of the interaction term was tested using the likelihood ratio test between the full model and the reduced model, which excluded the interaction fixed effect. The expectation–attention interaction was not significant for MSPCstim (χ2 = 3.47, *P* < 0.05) but was highly significant for MSPCres (χ2 = 19.56, *P* < 0.001). The data underlying this figure is available in FigShare at DOI: 10.26180/5b9abfe5687e3. MSPCres, multispectral phase coherency (response); MSPCstim, MSPC (stimulus).

The interaction between expectation and attention was not significant for MSPCstim (χ2 = 3.47, *P* > 0.05), but it was indeed highly significant for MSPCres (χ2 = 19.56, *P* < 0.001) (Fig 5). In fact, the slope of MSPCres against expectation was negative for unattended images (χ2 = 5.05, *P* < 0.05). Additional posthoc analyses performed individually for expectation and attention (S2 Text) were consistent with the results from Experiment 1 and Experiment 2, for which MSPCstim showed greater enhancement with expectation and MSPCres showed greater enhancement with attention. These results are interpreted further in Discussion.

Finally, we performed several analyses to examine the relation between the MSPC and amplitude measures. Specifically, we examined whether reduced SWIFT, SSVEP, and/or IM response amplitudes can account for the reduction of MSPCres in the unattended compared to the attended conditions. While we expect the MSPC and amplitude measures can, in theory, be correlated to some degree, we confirmed empirically that the amplitude measures accounted for no more than 20% of the MSPC variance. Further details of the analyses are provided in S1 Text.

## Discussion

The goal of this study was to examine the mechanisms underlying attention and expectation in perception, focusing on their modulation of top-down and bottom-up signal integration. All experiments utilised the HFT method [22] in which SSVEP- and SWIFT-tagged signals reflect activity at lower (V1/V2) and higher levels of the visual hierarchy, respectively [25,27]. Importantly, IM frequency components are a distinct and objective indicator for nonlinear integration of multiple input frequencies. Accordingly, we hypothesised the IMs here to be influenced by, and provide an indication of, the level of integration between top-down, semantically rich SWIFT-tagged signals and bottom-up, SSVEP-tagged sensory signals (see Introduction).

Two new experiments and a reanalysis of an existing data set with two novel phase-based measures (MSPCres and MSPCstim) were examined in this study, covering a range of experimental modulations of expectation and attention. The first experiment modulated expectation while keeping the motivational (task) relevance constant, and the second experiment modulated attention while holding expectation levels constant. Critically, each experiment used the same type of sensory input across the different conditions within the experiment, while cognitive modulations were achieved only by means of the behavioural tasks at hand. Our results provide direct neural evidence for the increased integration of bottom-up and top-down signals through modulation of expectation and attention.

Importantly, we argue that the dissociation between the MSPCstim and MSPCres measures found here relates expectation and attention to distinct mechanisms impacting the integration of descending and ascending signals at lower and at higher levels of the visual hierarchy, respectively.

Our results lend strong support to models of perceptual inference that emphasise the role of recurrent networks and the bidirectional flow of both bottom-up and top-down signals. We believe this study provides the first direct demonstration of the different neurophysiological manifestations of the mechanisms by which expectation and attention change the very integration of such top-down signals with bottom-up signals in perception.

As we show below, among the various models of perception that assume recurrent neural architectures, those predictive coding models that postulate expectation as top-down signals and attention as a mechanism for modulating bottom-up signals (Fig 1A) provide a particularly suitable framework under which our results can be interpreted.

### Terminology and theoretical background

The terms expectation and attention have been confounded in various cognitive studies (for discussion, see [8]). Furthermore, both attention and expectation are composed of many different aspects (e.g., spatial- versus feature-based attention, selective versus sustained attention, etc. Expectation can result from implicit learning of statistical contingencies or through explicit instruction, etc.), which can have distinct neural origins and underlying mechanisms. Like Summerfield and colleagues [8], we use the term attention to denote the motivational relevance of an event and the term expectation to denote its conditional probability.

The terms ‘predictions’ and ‘expectations’ can be used in the more technical predictive coding literature to denote somewhat different aspects of the perceptual circuitry. Predictions, in classical predictive coding models [2,11,37], are the descending signals that are used to explain away sensory or neural input in lower areas. Expectations on the other hand are described in some models as the inferences made about the state of the external world (or more technically speaking, the inferred values assigned by the generative model to the hidden causes and states in the external world). In that sense, predictions are compared against sampled sensory inputs in the sensory cortex or against expectations at intermediate hierarchical levels [3,20]. We use ‘expected’ and ‘predictable’ interchangeably here.

Attention, in the cognitive neuroscience literature, is commonly viewed as mechanisms which 1) increase baseline neural activity, sometimes referred to as arousal or alertness; 2) selectively enhance relevant neural responses; and 3) selectively inhibit irrelevant responses [38]. In Experiment 2, participants had to count only one of the two image categories in each trial. Here, we use the term ‘attended’ to refer to the counted object (a potential target for such attentional enhancement) and the term ‘unattended’ to refer to the object that was not counted (a potential target for such attentional inhibition). In some predictive coding models, such signal modulation is suggested to be achieved by means of internal estimates (and expectations) of precision (the inverse of signal variability). Greater precision estimates lead to greater weighting of the prediction error, effectively serving as a gain control for the bottom-up signals and allowing greater modification of higher-level expectations and predictions [39,40]. Attention, under this framework, enables signal enhancement by modulating the gain on the prediction errors obtained through varied estimations of prediction error precision estimations [3].

EEG signals are thought to be generated by the spread of postsynaptic potentials along the apical dendrites of pyramidal cells [41]. In other words, they are generated by activity at afferent rather than efferent pathways. The implications of this can be illustrated by assuming a process involving, for example, three hierarchical levels, with the lowest level sending bottom-up signals to the levels above, the highest level sending top-down signals to the levels below, and the middle level sending both bottom-up and top-down signals to the levels above and below, respectively. The lowest level in this case can be expected to primarily receive (and manifest the effects of) the top-down signals, while the highest level can be expected to primarily receive (and manifest the effects of) the bottom-up signals. We later argue that our results can be explained using such a simplified hierarchical structure in which expectation effects top-down signals while attention modulates the propagation of feedforward signals. (Later in this section, we show a simplified version of this with only two levels.)

### What can be learnt from the IM components?

As the IM components are key to our analysis, it is important to clarify several issues. The first issue relates to the dependency of the IM signals on two main factors: the amplitude and phase of the input signals and the specific mechanism of signal integration at hand. The former factor is straightforward, as changing the power or shifting the phase of the input can lead to a shift in output power or phase, respectively. Using IMs to infer something about the latter factor is, however, less trivial since nonlinear neuronal dynamics may be consistent with various models of neural processing, ranging from cascades of nonlinear forward filters (e.g., convolution networks used in deep learning) to the recurrent architectures implied by models of hierarchical perceptual inference [5,11,13]. It is therefore not easy to link specific computational or neuronal processes to the IM responses, and IMs can only provide indirect evidence for predictive coding as well as other theories of perception. However, as we will argue, various arguments indeed point to the recurrent and top-down mediation of the IM responses in our data.

The second issue to consider are the benefits afforded by the phase-based MSPC measure. For example, when using amplitude-based measures (power, etc.), one can only interpret changes in the IMs in respect to the fundamental frequencies (and their harmonics) since changes in the power of the fundamentals can lead to changes in the power of the IMs even without any essential changes in the integration process itself (for details, see [34]). It is therefore important when using amplitude-based measures to dissociate between the effects of the IMs and those of the fundamentals and harmonics (as we did in the previous study [22]). Conversely, the MSPC in itself quantifies the degree of temporal consistency of the relationship between the phases of the fundamental frequencies and the phase of the IMs. The MSPC can therefore be interpreted even if the amplitudes of the IMs and the fundamentals do not dissociate between conditions (see also the last part of Result).

In addition, unlike traditional amplitude measures, MSPC enabled us to make the novel distinction between MSPCstim and MSPCres, which was essential to this study. As mentioned, the MSPC measure aims to quantify the degree to which the phase of an IM component is driven by the phases of the input frequencies. The question then is what we consider those inputs to be. SSVEP and SWIFT phases can be quantified either by the stimulus itself or by the EEG response signal (as tagged and obtained by the FFT). We term these options MSPCstim and MSPCres, respectively. When considering these two measures, one may a priori expect them to behave quite similarly. After all, the stimulus (image) and the response (EEG) phases can be expected to be highly correlated with each other, leading to highly correlated MSPCstim and MSPCres measures. Therefore, our finding here is highly nontrivial; the expectation and attention manipulations influenced these measures in a highly different manner (Figs 3–5), directly indicating distinct neural processes for expectation and attention.

Our understanding of the IMs in this study as reflecting recurrent hierarchical interactions therefore stems from the combination of two main arguments. First, the SSVEP and SWIFT EEG signals have been shown to primarily originate from lower and higher visual areas, respectively (see Introduction). That said, the SSVEP and SWIFT signals likely travel across and may potentially interact at multiple hierarchical levels. As we describe in greater detail in Methods, we reason that since MSPCstim and MSPCres are computed based on the phases of the stimulus and the EEG response, respectively, MSPCstim most likely picks up interactions occurring at earlier stages than those picked up by MSPCres. Hence, while MSPCstim and MSPCres can in theory be correlated, our empirical findings across the three experiments strongly dissociated them, implying distinct underlying neural correlates spanning multiple hierarchical levels.

To account for these findings, we need a theoretical framework. As we propose in this paper, we find predictive coding to be particularly suitable for this matter.

### Expectation and attention: Main effects and their interaction

Our results demonstrate that expectation and attention reflect distinct elements of the perceptual circuitry. Combining results from all experiments, we show that the MSPCstim and MSPCres measures are more strongly related to the expectation and to the attention modulation, respectively.

**Fig 6:**
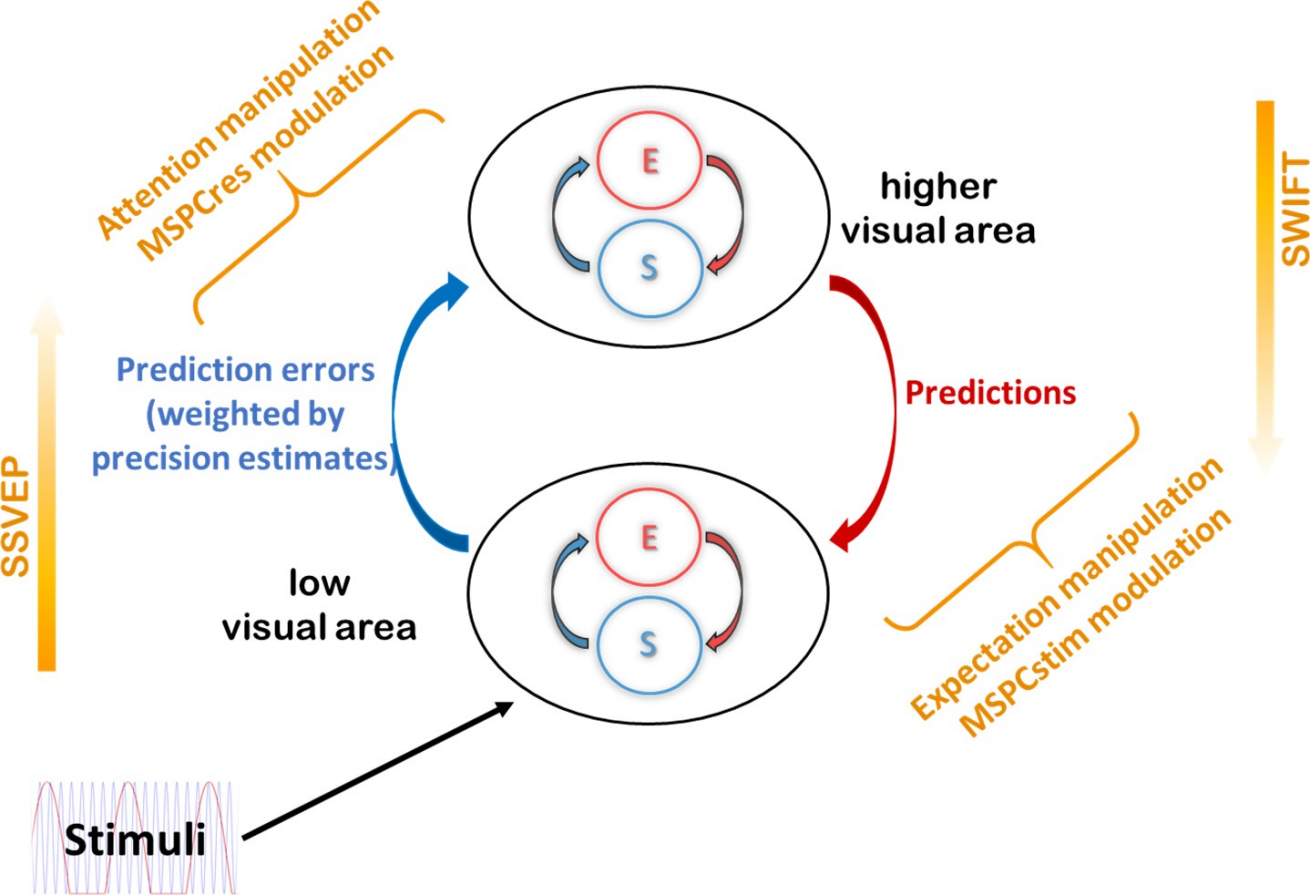
Expectation, attention, MSPC, and predictive coding. Results presented in this study can be accounted for by the predictive coding framework of perception as follows: 1) Expectation (the probability for the appearance of specific stimuli) relates to descending prediction signals. 2) Better predictions (as afforded by the PV trials in Experiment 1) increases the efficiency of top-down and bottom-up signal integration at low-level visual areas, as reflected by the increased MSPCstim with expectation (Figs 2 and 4). 3) Attention reflects a (precision-weighted) control mechanism for the propagation of prediction error signals. 4) Attention effectively increases the influence of prediction error signals on expectations at higher hierarchical levels, as reflected by the increased MSPCres with attention (Figs 3 and 4). 5) The effect of expectation on the integration of top-down and bottom-up information at lower visual areas is less dependent on attention than the integration at higher levels. Hence, while MSPCstim increased with expectation for both attended and unattended stimuli (Fig 5), the influence of expectation on MSPCres was attention dependent (Fig 5). MSPCres, multispectral phase coherency (response); MSPCstim, MSPC (stimulus); PV, pattern violation.

The MSPCstim measure demonstrated a consistent increase with expectation, as evident in the data obtained from Experiment 1 and from Gordon and colleagues [22]. Describing a stimulus as being predictable implies that the prediction signal precedes the onset of the stimulus itself. Hence, when the sensory input arrives, the prediction can be tested against (interact with) the incoming sensory-driven information in a highly ‘online’ manner. In such conditions, as in the PV trials in Experiment 1 and the ‘high-certainty’ trials by Gordon and colleagues [22], top-down predictions and bottom-up sensory evidence can interact quickly at early visual areas, and the resulting IM phase can be expected to be strongly related to the stimulus phase (see suggested primary source of MSPCstim in Fig 6). This observation is consistent with various studies demonstrating effects of expectation at early visual areas [42–44].

The MSPCres measure, on the other hand, showed a greater increase with attention in Experiment 2 and the Gordon and colleagues data compared to the MSPCstim, with no consistent modulation by expectation. Here, we have suggested MSPCres to be more strongly related to signal integration occurring at higher levels of the visual hierarchy. Attention is generally viewed as a mechanism that constrains the processing and propagation of bottom-up signals [14]. This idea has been incorporated into some predictive coding models by suggesting that attention optimises perception by allowing the ascending error signals to exert a greater influence on the expectations at the higher cortical level [40]. Under such a framework, attended (compared to unattended) images should allow greater integration of bottom-up error signals with higher-level expectations, as reflected by the MSPCres (see suggested primary source of MSPCres in Fig 6). Additional support for this claim is provided by the MSPCres analysis of the fourth-order IMs, which suggests that the modulatory effect of attention involves interactions occurring at a later stage than initial sensory processing (see S3 Text and S2 Fig).

In addition, an interesting relationship between expectation and attention follows the account of precision-weighted prediction errors (Fig 1A). On the one hand, highly predictable stimuli will yield small prediction errors. On the other hand, if a stimulus is highly relevant (attended), the influence of the prediction errors on the higher-level expectations will be enhanced [40]. Indeed, the significant interaction found for the MSPCres measure (Fig 5, right panel) supports such a relationship between expectation and attention. When stimuli are expected, predictions become more accurate, and their integration with sensory evidence at low visual areas improve. Hence, the MSPCstim response, which we suggest reflects neural integration at early visual areas, is enhanced with expectation, regardless of attention. In turn, when stimuli are attended, the propagation (or influence) of ascending error signals to (or on) the higher hierarchical level is enhanced. Hence, the MSPCres response, which we suggest reflects neural integration at later visual areas, is enhanced with attention. In contrast, when the expected stimuli are unattended (not task relevant), not only are the prediction errors gated out by (un)attention, they are also smaller to begin with, hence the reduced MSPCres.

### Questions for future research

Our results, we argue, empirically support two main ideas. First, that perception is realized by a recurrent hierarchical neural network in which interactions between bottom-up and top-down signals play a crucial role. Second, that these interactions are influenced by expectation and attention which reflect intrinsic yet distinct components of the perceptual network.

We further proposed a more fine-grained interpretation of our results by appealing to the free energy predictive coding model of perception and attention as formulated by Feldman and Friston [3]. We appeal to this particular model as it explicitly incorporates expectation and attention in a manner consistent with our results. Nevertheless, additional models of hierarchical perceptual inference incorporate interactions between top-down and bottom-up signals and, as such, should be considered as well.

Other Bayesian models such as that proposed by Lee and Mumford [5] also emphasize the critical functional roles of top-down probabilistic priors fed-back from the higher to lower hierarchical levels and, as such, can also account for the expectation-dependent integration of top-down and bottom-up signals, which we suggest here to be reflected by the IMs. Attention, however, is formulated in Lee and Mumford’s model as a ‘subset’ of such top-down priors, rendering the model less capable of accounting for the dissociation between expectation and attention found here.

In the predictive coding model proposed by Spratling [12], predictions exert their effect on the hierarchical level below, while object-based attention exerts its effect on the higher hierarchical level. This distinction is consistent with our interpretation of the MSPCstim and MSPCres measures and their distinct modulation by expectation and attention. Nevertheless, Spratling’s model [12] may be less suitable to account for the expectation–attention interaction shown in Fig 5.

Non-Bayesian models of hierarchical perceptual inference may also be consistent with our association of the IMs in the HFT paradigm with the integration of higher-level, more abstract, and semantically meaningful signals, with lower-level features extracted from the sensory input. Such a representational approach is set forward, for example, in the structural coding theory of perception [45], which also incorporates task-relevant attention into its view of hierarchical visual processing. Nevertheless, in structural coding, top-down signals (or hypotheses) are understood to be constructed on the fly from the sensory data, rather than reflecting expectations derived from past experience. It is therefore less clear how well structural coding lends itself to explaining the effect of Experiment 1 in which the prior expectations are not directly related to the complexity of the stimuli.

While we find the predictive coding model set forward by Feldman and Friston [3] particularly capable of accounting for our results, our experiments were not designed specifically to validate or test aspects of the model such as precision weighting.

Alternative explanations, unrelated to predictive coding, should be considered as well. One possibility is that some of the differences between the MSPCstim and MSPCres responses may be attributed to the locations of the neural generator sources mediating our expectation and attention modulations. For example, the posterior ROI used in our analyses may have captured attention-related parietal activity more so than expectation-related frontal activity.

Certainly, it would be interesting to see how additional modelling and theoretical studies may better explain our results and make further predictions about potential variants of the HFT method, the experimental paradigms used in this study, and the MSPC analyses we have implemented.

Another aspect that should be considered is the potential contribution of additional cognitive factors besides expectation in Experiment 1 and attention in Experiment 2. These may include, for example, differences in work load, spatial attention, and working memory in Experiment 1 or postperceptual counting effects in Experiment 2. In the current study, we use the term attention synonymously with task relevance, and the images presented in both tasks of Experiment 1 were indeed task relevant. Nevertheless, the different nature of the tasks can perhaps lead a participant to adapt different strategies. For example, they may pay more attention to the global aspects of the image in the PV trials and to the more local features in the IR trials.

These possibilities notwithstanding, there are several reasons to believe expectation is indeed the primary factor underlying the observed effects in Experiment 1. First, the modulation of the MSPCstim in Experiment 1 is consistent with the changes observed for the expectation factor in the analysis performed on the data from Gordon and colleagues [22]. There, a single behavioural task was used throughout the experiment. (Expectation was operationalised based on changes in the proportion of the images presented in each trial.) Second, the effect of attention in Experiment 2 and in the data from Gordon and colleagues [22] was fundamentally different and was manifested primarily in the MSPCres measure. These results suggest that it is very unlikely that the effects found in Experiment 1 can be explained solely by changes in the nature of attention between the PV and IR tasks. In addition, while all participants reported that the images in the IR trials were much less predictable than those of the PV trials, there were mixed reports about which of the tasks felt harder or more demanding. We did not formally collect the latter reports about the subjective level of difficulty from all participants so we cannot run a formal analysis to test the potential relationship between task difficulty and the modulation of the EEG measures; nevertheless, this may provide additional anecdotal evidence arguing against such dominant effects of workload, working memory, etc.

### Concluding remarks

Whether and how expectation and attention interact in perception is an ongoing debate in the scientific literature. Several studies have demonstrated a relationship between expectation and attention consistent with the interaction reported here. For example, using multivariate pattern analysis in fMRI, Jiang and colleagues [15] demonstrated that the ability to discriminate expected and unexpected stimuli was notably enhanced with attention. In a different fMRI study, Kok and colleagues [46] showed that a significantly reduced neural response to predicted stimuli was observed in V1 (but not in V2 and V3) only for the unattended stimuli. Attention, on the other hand, was shown to correlate with an enhancement of the forward drive of information from V1 to V2 and V3 and was therefore proposed to reflect an increase in the postsynaptic gain of prediction error neurons. Expectation–attention interactions have also been demonstrated in several EEG studies focusing on event-related potential (ERP) components occurring within 100–200 ms after stimulus onset [47].

Critically, the methods used in the current study avoid some of the principal limitations of those studies:

1) The effects of expectation and attention were not inherently confounded neither with each other (though see cautionary note above) nor by changes in low-level properties of the sensory stimuli. This was accomplished by designing the paradigms of Experiment 1 and Experiment 2 such that the cognitive domain was manipulated by changing the behavioural task at hand.

2) The primary variable used in our analyses provides a direct neural measure of signal integration within the context of hierarchical processing. This was accomplished by using the HF T method and by focusing analysis on IM components using the MSPCstim and MSPCres measures.

The above, with the combined results from multiple experiments, offer a unique advantage: the ability to obtain a direct and objective neurophysiological measure for the influence of expectation and attention on the integration of distinct streams of neural information in perception.

Instead of formulating the question at hand as whether expectation and attention increase or decrease neural activity, we place a spotlight on the role of signal integration in perception. In line with the predictive coding framework of perception, we view expectation and attention as distinct yet related mechanisms serving the common goal of approximating Bayes optimal perceptual inference. Our results highlight the role of feedback loops and integration of information across multiple hierarchical levels in the cortex and relate expectation and attention to descending and ascending signals, influencing information integration at lower and higher cortical levels, respectively.

## Methods

### Ethics statement

Experimental procedures were approved by the Monash University Human Research Ethics Committee (10994), which adheres to the Australian Code for the Responsible Conduct of Research, 2018. Participants gave their informed consent in writing to participate in the experiment.

### HFT

The HFT method is based on the combination of two frequency-tagging methods: a contrast modulation inducing SSVEP tagging activity at lower visual areas and an object-recognition modulation inducing SWIFT tagging activity at higher visual areas.

A detailed description of the method for creating SWIFT sequences can be found elsewhere [25]. The crux of the SWIFT method is that it scrambles contours while conserving local low-level attributes such as luminance, contrast, and spatial frequency. A SWIFT ‘sequence’ is a series of frames that, when presented sequentially, create a ‘movie’ that starts from a scrambled frame, progresses toward the middle (‘peak’) of the sequence where the original image can be briefly identified, after which the image becomes scrambled again. When such a SWIFT sequence is presented repetitively at a given frequency, the original images peaks at that frequency (i.e., once each SWIFT ‘cycle’), allowing the SWIFT tagging to be obtained (if the image is recognised).

To create a SWIFT sequence, we first scrambled the original image using the discrete Meyer wavelet and six decomposition levels. The local contour at each location and scale was then represented by a 3D vector. To create the sequence of frames, we then randomly selected two additional vectors of identical length and origin and defined the circular path that connected the three vectors (maintaining vector length along the path). We then performed additional cyclic wavelet-scrambling by rotating each original vector along the circular path and applying the inverse wavelet transform to obtain the image in the pixel domain. This way, we were able to smoothly scramble the original image by destroying contour information while conserving the local low-level attributes. The Matlab script for creating SWIFT sequences can be found at Koenig-Robert and colleagues [25].

In all experiments described here, we constructed each trial using SWIFT sequences created from one face and one house image, randomly selected for each trial from a pool of images. SWIFT sequences were presented consecutively, resulting in a ‘movie’ in which the original images (either the house or the face) were identifiable briefly around the peak of each such cycle (S1 Video).

The SWIFT method preserves the low-level local visual properties across all frames within each sequence (cycle). These properties could differ significantly between a pair of SWIFT sequences used within a single trial (one for a face and the other for a house). Therefore, to preserve the low-level local visual properties across the whole trial, regardless of the recognisable image in each cycle, it was essential to alpha-blend (with equal weights) frames from both the house and the face sequences. As described below, a single SWIFT frequency was used in Experiment 1 (S3 Video), with either the face or the house image appearing in each cycle. To allow only one image to be recognised in each cycle, we created additional ‘noise’ sequences and alpha-blended them with the frames of the ‘image’ sequences presented in that cycle. This was done in the following way: First, we selected one of the scrambled frames from each of the original SWIFT sequences (the frame most distant from the original image presented at the peak of the cycle). Then, we created noise sequences by applying the SWIFT method on the selected scrambled frame. In this way, each original ‘image’ sequence had a corresponding ‘noise’ sequence that matched the low-level properties of the image sequence. Finally, during the actual presentation of the stimuli, we alpha-blended (with equal weights) the frames of the ‘image’ sequence that appeared in that cycle with the ‘noise’ frames of the other image category (see Fig 2A at Gordon and colleagues [22]). In experiment 2, 15%–30% of ‘image’ cycles were replaced with ‘noise’ cycles to make the counting task attentionally demanding (S4 Video; see further details below). We performed this noise replacement separately for the face cycles and for the house cycles, which cycled at different frequencies (see below). This way, the overall low-level visual attributes were constant across all frames within the trial, regardless of the identifiable image in each cycle.

To minimise the possibility of confounding our results with the tagging of low-level visual features (specifically, by the cyclic repetition of the scrambled frames presented in between peaks) we created three sets of ‘image’ and ‘noise’ SWIFT sequence variants for each of the original images and continuously alternated between the sets during the trial. The timing of the transition between SWIFT variants (of a single original image) was designed to allow smooth transitions. This was done as follows: 1) If the image to be presented in the coming cycle is the same as the one presented in the current cycle (e.g., face-face), then the SWIFT variant of the ‘image’ sequence would be swapped at the peak of the current cycle (i.e., at the frame containing the original image). 2) If the image to be presented in the coming cycle is not the same as the one presented in the current cycle (e.g., face-house in experiment 1 or face-noise in experiment 2), the transition between the image and its matching noise sequences would occur at the frame from which the ‘noise’ sequence was created. This way, we could better control for potential tagging of low-level visual features while ensuring smooth transitions between cycles.

For SSVEP, a global sinusoidal contrast modulation was applied on the whole movie. To avoid both excessively strong SSVEP signals and total blanks of the SWIFT sequences, the contrast modulation was limited to 70% of the full contrast range (from 30% to 100% of the original image contrast) (S2 Video).

### Participants and experimental procedure

All participants were university students aged between 18 and 34 years. A total of *n* = 15 out of 24 participants were included in the analysis of Experiment 1, and *n* = 11 out of 16 in Experiment 2. Exclusion criteria were based on EEG quality and on task performance (see below.) In all experiments, participants were comfortably seated in a dimly lit room 55–60 cm in front of the monitor (LCD, 120 Hz refresh rate). Stimuli were presented at the centre of the screen over a grey background, and participants were asked to keep their fixation at the centre of the display. Participants were asked to minimise blinking or moving during each trial but were encouraged to do so if needed in the breaks between trials. Trials always began by the participant pressing the space bar.

Importantly, both new experiments performed in this study were designed such that the high-level factor was manipulated without modifying the stimuli used across experimental conditions. In other words, the predictability of the stimuli in the Experiment 1 and the relevance of the stimuli in Experiment 2 were manipulated through task instructions, without any confounds introduced by changes in low-level visual features.

### Behavioral tasks

#### Experiment 1: Expectation

Images appeared at 1.2 Hz, alternating between a face and a house image in specific orders. In each trial, participants were required to perform one of two tasks such that each task was performed in half the trials.

In the IR task, participants were asked to press the space bar when they identified any image repeating itself either three or four times in a row. The specific number (three or four) was displayed on the screen prior to each trial. For example, for a trial in which the participant was instructed to look for an image repeating itself three times in a row, the face (F) and house (H) images may have been presented in the following order:

F, H, H, F, F, H, F, H, H, F, F, H, F, H, H, F, F, **F**…

The ‘target’ image would then be the eighteenth image (F shown in bold). Throughout the paper, these trials are referred to as the IR trials.

In the PV task, participants were provided a pattern describing a series of 5–6 images prior to each trial and were instructed to memorise the pattern well. Participants were told that the images presented in the trial would follow the given pattern, which will repeat itself over and over again. The task was then to press the space bar as soon as they identified an image that violated the expected pattern.

For example, the following pattern may be verbally presented on the screen before a trial:

                     ‘Face, House, House, Face, Face, House’

After memorising the pattern, participants would begin the trial by pressing the space bar. Images would then appear in the following order:

F, H, H, F, F, H,        F, H, H, F, F, H,        F, H, H, F, F, **F**…

The ‘target’ image in this case would be the eighteenth image shown in bold. The spaces between each pattern in the above example are provided here for illustration alone. In the actual trials, images appeared consecutively as per the 1.2 Hz SWIFT frequency. Throughout the paper, these trials are referred to as the PV trials.

As can be seen, the sequence of images and the target image are identical in both above examples, allowing differences in conscious perception, behavioural performance, and evoked neural activity to be attributed strictly to the task-related requirements rather than the visual stimuli. We hoped that participants would not be aware of the underlying pattern when performing the IR task, rendering the series of images appear more random. As detailed in Results, this was indeed the case.

A global SSVEP contrast modulation was applied at 15 Hz in all trials.

After 1–2 training trials, four 11-trial blocks were administered in the following order: PV block, IR Block, PV block, IR block. Every series that appeared in the PV block was repeated in the following IR block in a random order of trials, using a different set of face and house images. PV and IR trials were then analysed as paired.

Within 3 minutes from completing the experiment (i.e., after the end of the fourth block), participants were asked to compare the difficulty level between the PV and IR tasks and to report whether they had noticed the underlying patterns in the IR tasks.

#### Experiment 2: Attention

HFT trials were constructed using house and face images. Contrary to Experiment 1, here, the two image categories were presented at different SWIFT frequencies. In other words, the house and face images each cycled at its own frequency (0.8 Hz and 1 Hz, counterbalanced.) (S4 Video). Each SWIFT frequency interacted independently with the SSVEP frequency, yielding its own set of IMs. Therefore, in Experiment 2, we had two second-order IMs (SSVEP + SWIFT) for each SWIFT–SSVEP combination. For example, in trials for which the SWIFT frequency of the attended image was 0.8 Hz, that of the unattended image was 1 Hz and the SSVEP frequency was 12 Hz. In those cases, the second-order IMs 11.2 Hz and 12.8 Hz indicated an interaction between the SSVEP and the SWIFT of the attended image, while the second-order IMs 11 Hz and 13 Hz indicated an interaction between the SSVEP and the SWIFT of the unattended image. This allowed us to separately tag the EEG responses associated with the recognition of each image.

Participants were instructed before each trial to count one of the image categories (e.g., ‘count houses’). Each image was therefore considered attended or unattended depending on what the participant was instructed to count during the trial. To ensure participants were actively paying attention to only one of the categories, rather than just following a certain ‘rhythm’, images were presented in only 70%–85% of their respective cycles and were substituted with their matching ‘noise’ sequence in the remaining cycles (S4 Video). Trials were 31.5 seconds long, allowing the total amount of counted images to range from 17 to 26 images per trial. Participants were instructed not to expect trials with less than 10–15 images and were requested to be as accurate as possible when counting.

To further verify the dependence of the SWIFT response on attention, we added a third condition in which participants were required to perform a demanding central-attention task, leaving only minimal spatial attention elsewhere on the screen [48,49]. In this task, participants were instructed to attend to a central cross that varied in the height of the horizontal line (above or below the midline) and colour (red, green, or blue). The cross was updated at pseudorandom times, jittering over 850–1,000 ms to reduce frequency tagging by this stimulus (a constant time interval would yield an excessively strong tagging of the cross frequency). Participants were instructed to count the number of occurrences of two conjunction targets, defined as an upward and red cross or a downward and green cross. These central stimuli were also present in the count-face and count-house tasks, but they were irrelevant and ignored (S4 Video).

Ten trials were administered in a random order for each of the three behavioural tasks (counting houses, faces, or crosses), reaching 30 trials in total. The two images were presented at SWIFT frequencies of 0.8 Hz and 1 Hz, counterbalanced across trials. The global SSVEP contrast modulation was applied at 12 Hz.

### Data acquisition and processing

Data were collected at two facilities, both using BrainProducts 64 scalp electrode EEG systems. Data for half of the participants from Experiment 1 (*n* = 12) and all participants from Experiment 2 (*n* = 16) were collected using an active-electrode actiCHamp system. Data for the other half of Experiment 1 (*n* = 12) were collected using a passive-electrode BrainAmp-MR system (not within an MR environment). Continuous EEG was sampled at 1,000 Hz for all participants.

Data processing was performed using the EEGLAB toolbox [50] in MATLAB. All data were resampled to 500 Hz. A high-pass filter was applied at 0.3 Hz and data was converted to average reference after replacing noisy electrodes. To define noisy electrodes, each sample point was regarded as being noisy if it was either greater than +80 μV (or lower than −80 μV), contained a sudden fluctuation greater than 30 μV from the previous sample point, or if the signal was more than ±5 STD from the mean of the trial data in each channel. Cycles in which over 2% of sample points were noisy were regarded as noisy cycles. Channels were replaced using spherical spline interpolation if they were considered noisy in over 10% of cycles. An additional CleanLine procedure was then applied to reduce AC power artefacts around 50 Hz and 100 Hz. The CleanLine plugin for EEGLAB (Mullen, 2012. Available online at http://www.nitrc.org/projects/cleanline) reduces sinusoidal (line) noise while avoiding typical phase distortions that can be caused by notch filters.

### Exclusion criteria

Exclusion criteria were defined based on the quality of the EEG recording and the behavioural results. For the former, individual trials were marked for exclusion if over 10% of channels were considered noisy in that trial after preprocessing (as described above). Participants with over 20% of bad trials were excluded from the analysis. A total of five participants from Experiment 1 and four participants from Experiment 2 were excluded based on these criteria for poor EEG recordings.

To ensure participants were sufficiently engaged with the tasks, we excluded participants whose responses were considered invalid in over 30% of trials. For Experiment 1, responses were considered invalid if the space bar was not pressed during the trial or if it was pressed before the appearance of the target image (this criterion was applied on the PV trials). For Experiment 2, responses were considered invalid if they differed by more than ±3 from the correct number (i.e., the actual number of times the attended image appeared in the trial). A total of four additional participants from Experiment 1 and one additional participant from Experiment 2 were excluded based on these criteria for poor response accuracy.

### Spectral analysis

EEG amplitudes and phases were extracted for each trial at the tagging and intermodulation frequencies by applying the FFT over a predefined subset period. For Experiment 2, the FFT was applied on the 25-second epoch ranging from 3–28 seconds from trial onset, yielding a half-bandwidth of 0.04 Hz (= 1/25 s) (12,500 sample-points). For Experiment 1, we applied the FFT on either 10 or 20 seconds of data, depending on the amount of data available in each trial (trial lengths in this experiment varied according to the location of the target image and the participant’s response). To reduce onset effects and the nosier signals often seen near trial onsets, we excluded the first SWIFT cycle of each trial from all analyses. Trials with more than 17 seconds of available data were zero-padded to 20 seconds and analysed as a 20-second trial, with a half-bandwidth of 0.05 Hz (= 1/20 s). Trials with less than 17 seconds of data (but more than 10) were analysed using the first 10 seconds, with a half-bandwidth of 0.1 Hz (= 1/10 s).

SNRs were computed by dividing the amplitude at any given frequency by the arithmetic mean amplitude across its neighbouring frequencies [51,52]. The specific number of neighbouring frequencies used for the SNR calculation depended on the length of data used in each analysis (as described above), ranging from 4 on each side (from f − 0.4 Hz to f + 0.4 Hz) for the 10-second epochs to 8 on each side (from f − 0.32 Hz to f + 0.32 Hz) for the 25-second epoch. Any neighbouring harmonic or IM frequency falling within that range was removed from the SNR calculation.

While a theoretically limitless number of IM components exist (all combinations of non-zero integer-multiples of the fundamental input frequencies: *n*1f1 + *n*2f2, *n* = ±1, ±2, ±3…), we focused our primary analyses on the two lowest (2nd) order components (f1 ± f2), which tended to have the highest amplitude SNRs (as in [22]). Additional analyses were then performed on third- and fourth-order IMs to broaden the scope of our investigation, as described in greater detail in S3 Text.

### MSPC

Distinct aspects of nonlinear interactions may be revealed by examining both phase and amplitude information. Applying phase analyses rather than amplitude analyses alone may have several advantages. First, amplitude and phase information may indicate different aspects of neural processing, with the phase-coherence believed to reflect the relative timing of neural activity [53]. Second, noise that is not associated with stimulus processing is (by definition) not time-locked to stimulus onset and is therefore not expected to demonstrate any phase consistency across trials. Consequently, phase analyses may be more robust to noise, potentially allowing the detection of genuine response components even when the amplitude is low.

The phase-coupling measure we use in this study is the MSPC introduced by Yang and colleagues [36]. The MSPC is especially useful for the study of IM components since rather than comparing a frequency phase to a time-locked event, it allows it to be compared against the phases of the fundamental frequencies. In that sense, instead of asking ‘how consistently is the IM phase related to a given event in time’ (as with the classic phase-locking factor), the MSPC asks ‘how consistently is the IM phase related to the phases of the input frequencies for the IM’. The MSPC can be calculated between input (I) frequencies f_1_,f_2_ …f_r_ and any given output (O) IM (or harmonic) frequency $f_{Ʃ}=\sum_{r=1}^{R}n_{r}f_{r}$ (in which *f*_Ʃ_ is the sum of the non-zero integer-multiples of the input frequencies with weights *n*_1_, *n*_2_ …*n*_r_). Mathematically, this is given by the formula

$$\Psi_{IO}=\left|\frac{1}{k}\sum_{k=1}^{k}e^{i(\sum_{r=1}^{R}n_{r}\emptyset I_{k}(f_{r})-\emptyset O_{k}(f_{Ʃ}))}\right|$$

in which ∅*I_k_*(*f_r_*) is the phase of the input frequency *f*_r_ (in epoch k), and ∅*O_k_*(*f*_Ʃ_) is the phase of the output IM (in that same epoch).

**Fig 7:**
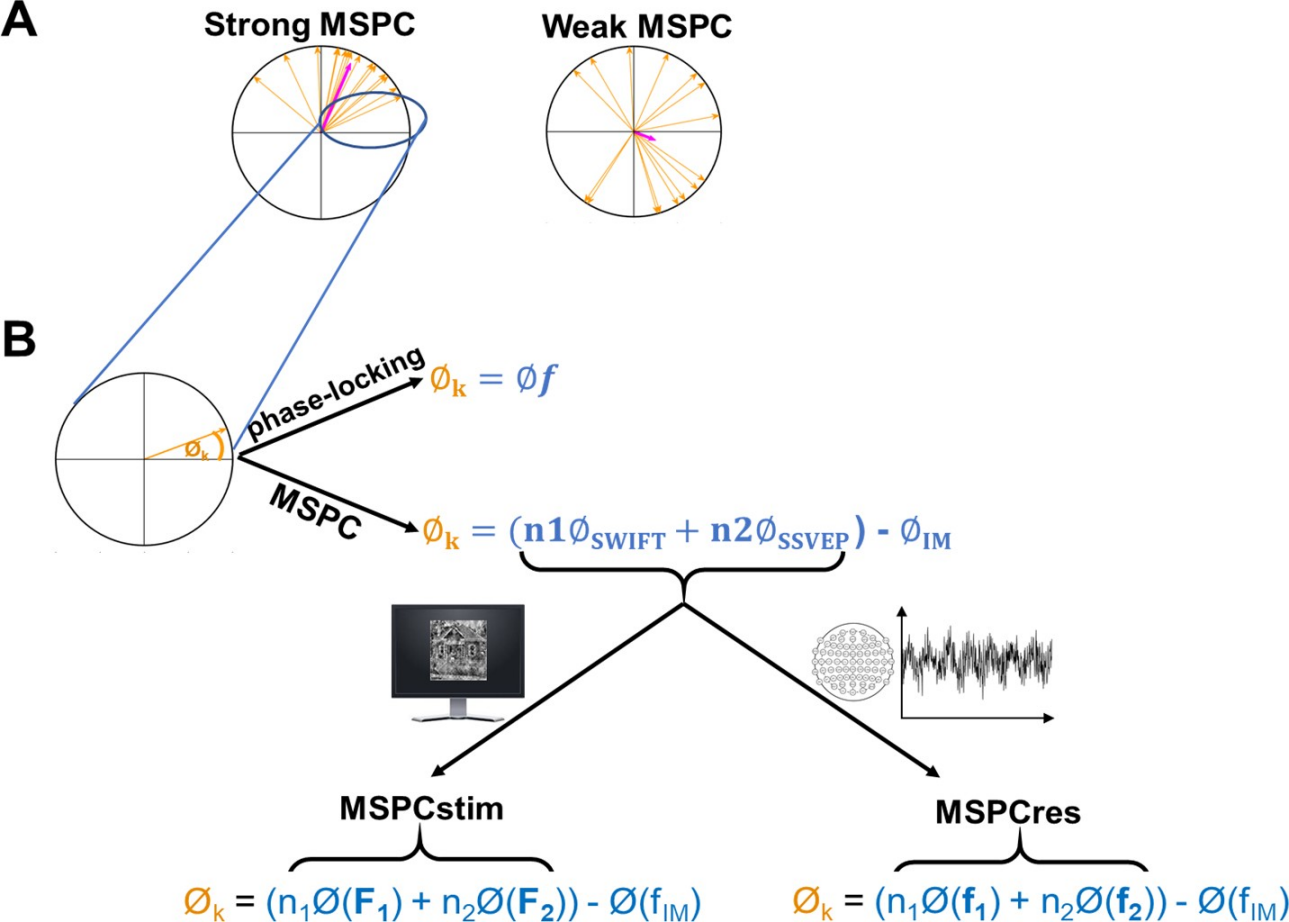
Multispectral phase coherence. The method for calculating MSPC. (A) Schematic example of stronger (left) and weaker (right) phase coherence. Both the PLV and the MSPC measures examine the consistency of a given phase term across multiple epochs. This can be visualised by first converting the phase term from each epoch into a unit (length = 1) vector pointing at its phase angle. Phase coherence is then obtained by computing the average vector (the sum of all vectors divided by the number of epochs). The result is a vector whose length can vary from 0 (each vector pointing at random directions, no phase consistency across epochs) to 1 (all vectors pointing at the same direction, perfect consistency across epochs). (B) The primary difference between PLV and MSPC measures is the phase term used for each epoch. For the PLV, only the phase of one specific frequency (or frequency band) is extracted for each channel/epoch and is used as the phase term for computing phase locking. When examining consistency between distant channels, the phase term used for the PLV would be the difference (Δφ) between the phases extracted from the different channels. In contrast, the phase term used for the MSPC calculations here is based on the difference (Δφ) between the phase of a specific IM component and the (weighted sum of) phases of the fundamental frequencies within each channel/epoch. In other words, the MSPC can be understood as a measure of the extent to which the IM phases are driven by the phases of the fundamental frequencies. Our distinction between MSPCstim and MSPCres is reflected by the two MSPC formulas shown at the bottom of the figure. Specifically, MSPCstim defines the phases of the stimuli (the on-screen image) as the input (or ‘driving’) fundamental frequencies (left formula, upper case F1 and F2), and MSPCres defines the EEG response phases as the input fundamental frequencies (right, lower case f1 and f2). Note that the weights of the fundamental frequencies in those formulas (*n*_1_ and *n*_2_) are the coefficients that define the IM frequency (e.g., given F1 = 1.2 Hz and F2 = 15 Hz, the weights for the third-order IM component 2*F1 + F2 = 17.4 Hz would be *n*_1_ = 2 and *n*_2_ = 1). EEG, electroencephalography; IM, intermodulation; MSPCres, multispectral phase coherency (response); MSPCstim, MSPC (stimulus); PLV, phase-locking value.

There are various additional conceptual differences between the more commonly used phase-locking value (PLV) and the MSPC measure we apply here. First, for PLV, the phase of only one frequency of interest is extracted from each epoch to calculate PLV values. Here, for each MSPC calculation, we extract the phases of three frequencies from each epoch: two fundamental and one IM component phase (Fig 7). Second, PLV is often used to examine the coherence between different neural signals by testing the consistency of the difference (Δφ) between phases of distant channels. Here, we test the consistency of the difference between the phases of the fundamental frequencies and the IM component within a channel.

The logic behind performing within-channel calculations is that the sources associated with the SWIFT, the SSVEP, and the IM responses should, in principle, have an additive (and therefore separable) influence on the resulting EEG signal at each channel. Moreover, if we assume that for any given channel, the phase of the signal associated with the activity of a particular source depends on a characteristic time delay, then the dynamics of a single electrode should contain information about the dynamics of multiple electrodes [54]. Therefore, we used within-channel data to examine the relationship between the IM and the SWIFT and SSVEP phases while avoiding the complexity involved in cross-channel computations.

A novel distinction we introduce here is between the calculation of the MSPC based on the stimulus and the response (Fig 7). We define the stimulus phase as the phase of the SSVEP contrast modulation and the phase of the SWIFT sequence relative to the original image. We define the response phase as the phases of the FFT of the EEG at the relevant frequencies (i.e., the SWIFT and the SSVEP frequencies). MSPC values were calculated for each channel individually.

We reasoned that separately considering the stimulus phase and the response phase as the ‘inputs’ of the IM response may allow a distinction between interactions occurring at lower and higher levels of hierarchical processing, respectively. The logic behind this is as follows: while the stimulus phases should relate consistently to the phases of low-level retinal signals, the response phases reflect activity occurring farther up the visual pathway. Importantly, however, the primary sources of the SSVEP and SWIFT EEG signals are not the same but rather low and high visual levels, respectively [25]. If the processing time delays between retinal input and activity at SSVEP-generating regions and SWIFT-generating regions were different, yet constant, the MSPC measure should not differ when calculated based on the stimulus or based on the response phases of the SSVEP and SWIFT. This is because MSPC measures the degree of consistency between an IM frequency phase and the combined SSVEP and SWIFT phases across epochs, which should not depend on the addition of constants. However, the processing times leading to activity at SSVEP- and SWIFT-responsive regions may not be constant across all stimuli. For example, the timing can vary depending on factors such as expectation, attention, image visibility, recognition, etc. Therefore, when examined across multiple epochs, the IM response phase may relate differently to the SSVEP and SWIFT stimulus phases than to the SSVEP and SWIFT response phases. This is the logic behind our assumption that these two measures, which we denote MSPCstim and MSPCres, may reveal different information about interactions occurring at lower and at higher levels of the visual hierarchy, respectively.

MSPC measures were calculated in a within-trial manner by dividing each trial into a series of 5-second (Experiment 1) or 10-second (Experiment 2) epochs, with a 1-second step from epoch to epoch. (Shorter epochs were used for Experiment 1 since the average length of data available for each trial was shorter than those of Experiment 2).

### Expectation–attention interaction (reanalysis of eLife)

In addition to the two experiments described above, new analyses were performed on the data published in Gordon and colleagues [22]. A detailed description of the paradigm and analysis methods can be found in the original publication. In brief, house and face SWIFT cycles were presented in each trial in a pseudorandom order and participants were asked to count either the houses or the faces. Certainty (expectation) levels were categorised based on the proportion of house and face images appearing in each trial and ranged from low certainty (faces and houses appeared at nearly 50% of cycles each) to high certainty (one of the two images appeared in nearly 100% of cycles).

Importantly, while the behavioural task allowed us to verify that participants were engaged with the task, it introduced a within-trial difference between the attended (counted) and unattended images, which was not analysed in that study. Given that one underlying SWIFT frequency was used (each cycle peaking at either the face or the house image) and the spectral analysis was performed on full trials, face- and house-driven EEG responses could not be distinguished from each other in the frequency domain. The effect of expectation on amplitude SNRs was examined in the original study using the linear mixed-effects (LME) analyses with a model that included certainty as the fixed effect and channels nested within participants as random effects.

Here, we first calculated new MSPCstim and MSPCres measures for the second-order IM components. Then, to allow the additional examination of potential interactions between expectation and attention (counted versus uncounted images), an additional attention-dependent variable was added: the attentional category of the more frequent (higher-certainty) image. In other words, this variable indicated whether the image presented in most cycles (above half) in any given trial was the counted or the not-counted image. This way, we could now distinguish between the effects of high expectation for attended versus for unattended images. In the new LME model, expectation, attention, and an expectation–attention interaction were included as the fixed effects. Random effects included a random intercept for frequency nested within channels nested within participants and random expectation, attention, and interaction slopes for each participant.

To examine the consistency of the full interaction LME model with the results from Experiment 1 and Experiment 2, we performed additional posthoc tests to individually examine expectation and attention. For conditions similar to those of Experiment 1, we first tested the effect of expectation within the attended condition. Then, for conditions similar to those of Experiment 2, we used a median split to reduce expectation to two bins (expected and unexpected), and we tested the effect of attention within the expected condition.

As in the original study, we tested for the significance of a given factor or interaction by performing a likelihood ratio test between the full model, as described above, and the reduced model, which did not include the factor in question [55].

## Supporting information

S1 Fig Amplitude SNRs demonstrate successful frequency tagging in Experiments 1 and 2. Results of the FFT averaged across all electrodes, trials, and participants. Amplitude SNR peaks can be seen at the tagging frequencies (solid lines) and their harmonics (dashed lines) in both Experiment 1 (A) (SWIFT: 1.2 Hz and SSVEP: 15 Hz, *N* = 15) and Experiment 2 (B) (two SWIFT: 0.8 Hz and 1 Hz and SSVEP: 12 Hz, *N* = 11). Note that in Experiment 2, no SWIFT tagging was obtained when counting crosses. (The peak at approximately 1.08 Hz matches the average amount of time between cross presentations which was 925 ms.) The data underlying this figure is available in FigShare at DOI: 10.26180/5b9abfe5687e3. FFT, fast Fourier transform; SNR, signal-to-noise ratios; SSVEP, steady-state visual evoked potential; SWIFT, semantic wavelet-induced frequency tagging.
(TIF)

S2 Fig The fourth-order IMs reflect a late interaction (the second-order IM between the second harmonics). Additional analyses of the fourth-order IMs were performed to compare between two potential two-stage second-order sequences: nonlinear processing of each of the input signal followed by an interaction between the two (i.e., F2,F1 → 2f2, 2f1 → 2f2 + 2f1, left bars) or an interaction between the input signals followed by an additional nonlinear process (i.e., F2,F1 → [f2 ± f1] → 2[f2 ± f1], right bars). Higher MSPCres values were obtained for both the attended and the unattended images when defining the second harmonics of the SWIFT and SSVEP response frequencies as the driving input signals. This indicates that the fourth-order IMs are driven more by these second harmonics than by their second-order IMs. Furthermore, attention had a significantly greater influence on the degree to which the fourth-order IMs were driven by the 2f1 and 2f2 harmonics than by the f1 + f2 IMs. These results are consistent with the notion that the attention modulation influences processes occurring at later stages than where initial input processing and interactions occur. The data underlying this figure is available in FigShare at DOI: 10.26180/5b9abfe5687e3. IM, intermodulation; MSPCres, multispectral phase coherency (response).
(TIF)

S1 Text MSPC and amplitude measures. MSPC, multispectral phase coherency.
(PDF)

S2 Text Interaction of attention and expectation. (PDF)

S3 Text Higher-order IMs. IM, intermodulation.
(PDF)

S1 Video The SWIFT method. A slow-motion demonstration of two SWIFT 'cycles' presented sequentially. SWIFT, semantic wavelet-induced frequency tagging.
(AVI)

S2 Video The HFT method. An 8-second demonstration of the HFT method combining two SWIFT images cycling at 0.8 Hz and a global SSVEP contrast modulation at 6.5 Hz. HFT, hierarchical frequency tagging; SSVEP, steady-state visual evoked potential; SWIFT, semantic wavelet-induced frequency tagging.
(AVI)

S3 Video Experiment 1: Expectation. A demonstration of one 'Expectation' trial (Experiment 1). The same stimuli can be used in both the IR and PV tasks. For the IR task, participants in this example trial need to press the space bar when any of the images repeats itself three times in a row. For the PV task, before this example trial, participants are presented with the words 'Face, House, House, Face, House, Face' and are required to memorise the pattern well. During the trial, images appear according to this pattern, and the pattern repeats itself over and over. Participant needs to press the space bar the moment the pattern is violated (i.e., when a face appears instead of a house or vice versa). This occurs in this example trial in the second before last image. IR, image repetition; PV, pattern-violation.
(AVI)

S4 Video Experiment 2: Attention. A 10-second demonstration of one 'Attention' trial (Experiment 2). One face image and one house image cycle at different frequencies. Images in this trial appear in only 70% of their respective cycles. Participants are instructed before each trial to count either the face or the house image. After each trial, participants enter the number of images they have counted throughout that trial. Note that this demo is greyscale. In the actual trials, the central-fixation cross alternated between the colours red, green, and blue.
(AVI)

## Abbreviations

EEG: electroencephalography
ERP: event-related potential
FFT: fast Fourier transform
fMRI: functional magnetic resonance imaging
HFT: hierarchical frequency tagging
IM: intermodulation
IR: image repetition
MSPC: multispectral phase coherence
MSPCres: MSPC response
MSPCstim: MSPC stimulus
O: occipital
P: parietal
PO: parieto–occipital
PV: pattern violation
ROI: region of interest
SSVEP: steady-state visual evoked potential
SNR: signal-to-noise ratios
SWIFT: semantic wavelet-induced frequency tagging
