# Supplementary figures and images for: Expectation and attention increase the integration of top-down and bottom-up signals in perception through different pathways
Source: PLoS Biol. 2019 Apr 30;17(4):e3000233. doi: 10.1371/journal.pbio.3000233 (PMC6490885; doi:10.1371/journal.pbio.3000233)

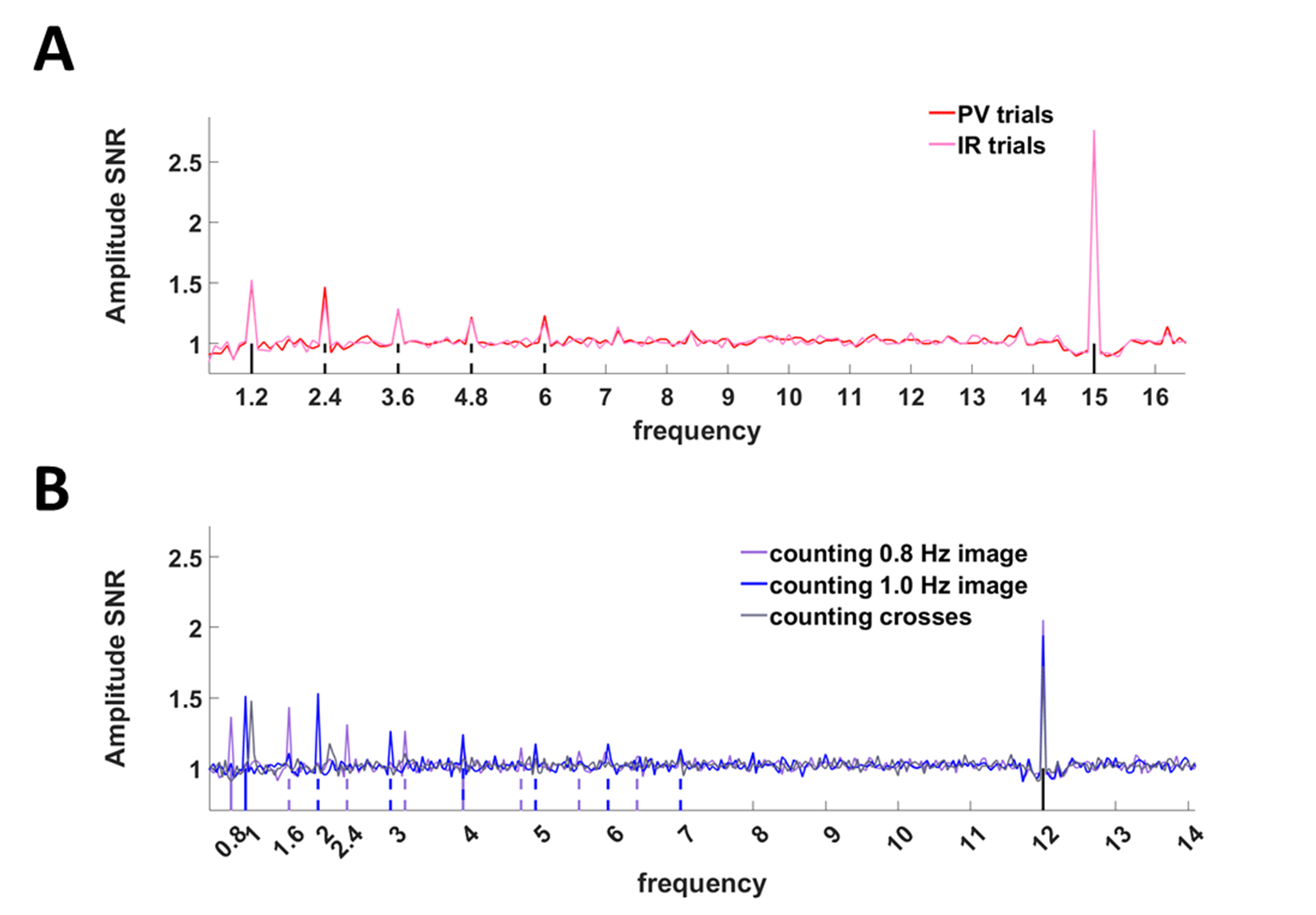

Supplement: S1 Fig — Results of the FFT averaged across all electrodes, trials, and participants. Amplitude SNR peaks can be seen at the tagging frequencies (solid lines) and their harmonics (dashed lines) in both Experiment 1 (A) (SWIFT: 1.2 Hz and SSVEP: 15 Hz, N = 15) and Experiment 2 (B) (two SWIFT: 0.8 Hz and 1 Hz and SSVEP: 12 Hz, N = 11). Note that in Experiment 2, no SWIFT tagging was obtained when counting crosses. (The peak at approximately 1.08 Hz matches the average amount of time between cross presentations which was 925 ms.) The data underlying this figure is available in FigShare at DOI: 10.26180/5b9abfe5687e3. FFT, fast Fourier transform; SNR, signal-to-noise ratios; SSVEP, steady-state visual evoked potential; SWIFT, semantic wavelet-induced frequency tagging. (TIF) [file pbio.3000233.s001.tif]

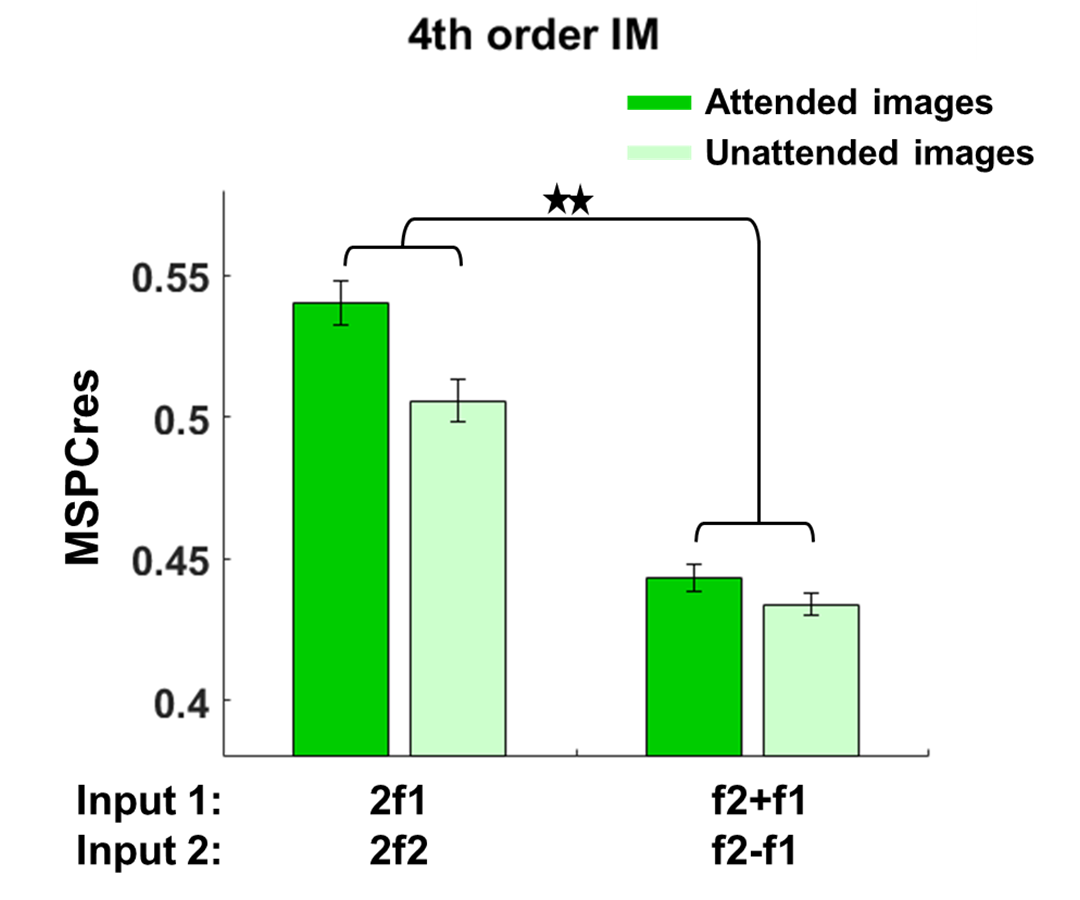

Supplement: S2 Fig — Additional analyses of the fourth-order IMs were performed to compare between two potential two-stage second-order sequences: nonlinear processing of each of the input signal followed by an interaction between the two (i.e., F2,F1 → 2f2, 2f1 → 2f2 + 2f1, left bars) or an interaction between the input signals followed by an additional nonlinear process (i.e., F2,F1 → [f2 ± f1] → 2[f2 ± f1], right bars). Higher MSPCres values were obtained for both the attended and the unattended images when defining the second harmonics of the SWIFT and SSVEP response frequencies as the driving input signals. This indicates that the fourth-order IMs are driven more by these second harmonics than by their second-order IMs. Furthermore, attention had a significantly greater influence on the degree to which the fourth-order IMs were driven by the 2f1 and 2f2 harmonics than by the f1 + f2 IMs. These results are consistent with the notion that the attention modulation influences processes occurring at later stages than where initial input processing and interactions occur. The data underlying this figure is available in FigShare at DOI: 10.26180/5b9abfe5687e3. IM, intermodulation; MSPCres, multispectral phase coherency (response). (TIF) [file pbio.3000233.s002.tif]
