# Supplementary material for: Expectation and attention increase the integration of top-down and bottom-up signals in perception through different pathways
Source: PLoS Biol. 2019 Apr 30;17(4):e3000233. doi: 10.1371/journal.pbio.3000233 (PMC6490885; doi:10.1371/journal.pbio.3000233)
Supplement: S1 Text — MSPC, multispectral phase coherency. (PDF) [file pbio.3000233.s003.pdf]

MSPC and Amplitude measures

We performed a series of analyses to examine the potential confound between the MSPC and amplitude measures. Specifically, given the significantly reduced SWIFT amplitude tagging for unattended images (S1 Figure), we wished to examine whether the reduction of MSPCres in the unattended images was a consequence of a potential decrease in the reliability of phase measures when amplitudes are low. For this, we performed additional analyses to evaluate the relation between MSPCres and the IM amplitude, and between MSPCres and the SWIFT amplitude (note that while different SWIFT frequencies were used in Experiment 2 for the attended and unattended images, only a single SSVEP frequency was applied, hence the SSVEP cannot be differentiated for the attended and unattended images in that experiment).

We performed all correlation analyses using the Spearman correlation within each participant. This way, we could avoid unwanted effects that can result from differences in the EEG topographies across participants or from the potentially different distributions of the MSPCres and amplitude measures. To do so, we first computed the MSPCres and the amplitude SNR for the second order IMs (f1+-f2) and the SWIFT frequency across trials for a given EEG channel for each participant. Then, for each participant we calculated the Spearman correlation between the MSPCres and the amplitude measures across all electrodes.

To examine the relationship between the MSPCres and the amplitude SNR of the *IMs* we performed two tests: 1) a correlation analyses between MSPCres and the amplitude SNR of the 2nd order IMs, and 2) a LME model (like the one used in Figure 5) with amplitude SNR as the predicted variable instead of the MSPCres. The results of the within-participant correlations analyses demonstrated low correlations between the MSPCres and the IM amplitude SNRs (average R^2=0.06, min R^2=0.00 and max R^2=0.22 across all participants). The results of the LME indicated that while MSPCres decreased with expectation in the unattended condition, the amplitude SNR in fact trended upward.

We also computed correlation between the MSPCres and the amplitude SNR of the *SWIFT* frequency. Also here, the within-participant correlations demonstrated low correlations (average R^2=0.04, min R^2=0.00 and max R^2=0.19 across all participants).

Taken together, these results indicated that the amplitude of the IM and SWIFT frequencies alone cannot account well for the behaviour of the MSPCres.
