# Supplementary material for: Expectation and attention increase the integration of top-down and bottom-up signals in perception through different pathways
Source: PLoS Biol. 2019 Apr 30;17(4):e3000233. doi: 10.1371/journal.pbio.3000233 (PMC6490885; doi:10.1371/journal.pbio.3000233)
Supplement: S2 Text — (PDF) [file pbio.3000233.s004.pdf]

Interaction of attention and expectation

After differentiating expectation and attention in Experiments 1 and 2, and establishing that both expectation and attention are associated with enhanced IMs, we returned to our previously published data (Gordon *et al.*, 2017) to examine the interaction between these factors, and to evaluate the consistency of the results from that study with those of Experiments 1 and 2 presented here.

In Gordon *et al.*, we analyzed frequency-domain amplitudes for SSVEP, SWIFT and IM, as a function of the number of counted stimuli, which confounded the effects of expectation and attention. Here, we separated the main effects of expectation and attention from their interaction within that single paradigm, using the MSPC-based measures as above. To do so, an additional 'attention' variable was added to the model, allowing us to examine the interaction between expectation and attention. The new 'attention' variable indicated, for each trial, whether it was the counted (attended) or the uncounted image that appeared in most cycles. Then, a likelihood ratio test was performed between the full LME interaction model (including expectation, attention and the expectation-attention interaction as the fixed effects) and the reduced model (including expectation and attention as fixed effects, without the interaction term) (see Methods).

The interaction between expectation and attention was not significant for MSPCstim ($\chi2 = 3.47$, $p>0.05$) but it was indeed highly significant for MSPCres ($\chi2 = 19.56$, $p<0.001$). In fact, the slope of MSPCres against expectation was negative for unattended images ($\chi2 = 5.05$, $p<0.05$) (Figure 5). These results are interpreted further in Discussion.

To examine the consistency of the results from this data with those of experiment 1 and 2, we performed individual post-hoc analyses for expectation and attention. For conditions more similar to those of experiment 1, we tested the effect of expectation within the attended condition. For conditions more similar to those of Experiment 2, we used a median split to reduce expectation into two bins (expected and unexpected) and we then tested the effect of attention within the high expectation bin. Consistent with the results from Experiments 1, the effect of expectation within the attended condition was significant for MSPCstim ($\chi2 = 7.35$, $p<0.01$) but not for MSPCres ($\chi2 = 1.86$, $p>0.05$), and the effect of attention within the expected condition was more notable for MSPCres ($\chi2 = 12.11$, $p<0.001$) than for MSPCstim ($\chi2 = 4.53$, $p=0.033$). The effect of attention on MSPCstim did not survive the false discovery rate (FDR) correction for multiple comparisons ($p= 0.066$ after FDR adjustment).
