# Supplementary material for: Expectation and attention increase the integration of top-down and bottom-up signals in perception through different pathways
Source: PLoS Biol. 2019 Apr 30;17(4):e3000233. doi: 10.1371/journal.pbio.3000233 (PMC6490885; doi:10.1371/journal.pbio.3000233)
Supplement: S3 Text — IM, intermodulation. (PDF) [file pbio.3000233.s005.pdf]

Higher order IMs

After establishing the modulatory influence of expectation and attention on the 2nd order IMs (f2$\pm$f1), we performed additional analyses on the 3rd (f2$\pm$2f1, 2f2$\pm$1f1) and 4th (2f2$\pm$2f1) order components. High-order IMs can arise from a sequence of lower-order computations. Here, we examined MSPCres at the higher-order IMs to compare between two plausible computational sequences.

Before doing so, we first examined whether the effects of the behavioural manipulations were evident in the 3rd and 4th order IMs. For experiment 1 (expectation modulation), only the 4th order IMs demonstrated similar modulatory influence of expectation, with MSPCstim of those IMs being higher in the PV (expected) compared to the IR (unexpected) trials ($\chi2= 8.04$, $p<0.01$). This effect was nevertheless not as significant as the effect observed for the 2nd order components ($\chi2= 22.9$, $p<0.001$). For experiment 2 (attention modulation), both the 3rd and the 4th order IMs demonstrated a similar modulatory influence of attention, with MSPCres being significantly higher for counted (attended) compared to non-counted (unattended) images ($\chi2 > 20$ and $p<<0.001$ for both comparisons).

The 4th order IMs can be described as the harmonic of the 2nd order IM (i.e. F2,F1 → (f2+f1) → 2(f2+f1)), or, alternatively, as the 2nd order IM between the harmonics of the fundamental frequencies (i.e. F2,F1 → 2f2, 2f1 → 2f2+2f1). To distinguish between these two possibilities, we ran additional MSPCres analyses in which we defined either the IM components f1+f2 and f1-f2, or the harmonics 2f2 and 2f1 as the driving input frequencies of the 4th order 2f2$\pm$2f1 IMs. In other words, we examined whether the 4th order IMs reflects ‘early-interaction’, driven by the 2nd order IMs (as would be the case if an initial interaction between the input signals is followed by another non-linear process) or ‘late-interaction’, driven by the harmonics of the fundamental frequencies (as would be the case if the input signals are processed individually and then interact).

For statistical analysis, we defined an LME model in which attention, the MSPC computation method (i.e. bases on 2f1 and 2f2 or based on f2-f1 and f2+f1) and the interaction between the two were included as the fixed effects. Random effects included a random intercept for frequency nested within channels nested within participants, and random attention, MSPC computation method and interaction slopes for each participant.

The MSPCres of the 4th order IMs was significantly higher when calculated based on the harmonics of the fundamental frequencies (2f1 and 2f2) than when calculated based on their 2nd order IMs (f2+f1 and f2-f1) ($\chi2= 27.2$, $p<0.001$) (S2 Fig). These results therefore favour the ‘late-interaction’ option in which

the input signals are processed individually before interacting with each other. Indeed, the interaction between attention and the MSPC computation method was significant ($\chi 2 = 8.4$, $p<0.01$), indicating that attention had a significantly greater influence on the degree to which the 4th order IMs were driven by the 2f1 and 2f2 harmonics than by the f1±f2 IMs. These results provide additional support linking the MSPCres measure and the attentional modulation to interactions occurring at later stages than where initial stimulus processing and interactions occurs.
